# Phenotypic Plasticity Regulates *Candida albicans* Interactions and Virulence in the Vertebrate Host

**DOI:** 10.3389/fmicb.2016.00780

**Published:** 2016-05-26

**Authors:** Emily M. Mallick, Audrey C. Bergeron, Stephen K. Jones, Zachary R. Newman, Kimberly M. Brothers, Robbert Creton, Robert T. Wheeler, Richard J. Bennett

**Affiliations:** ^1^Department of Molecular Microbiology and Immunology, Brown UniversityProvidence, RI, USA; ^2^Department of Molecular and Biomedical Sciences, University of MaineOrono, ME, USA; ^3^Department of Molecular Biology, Cell Biology, and Biochemistry, Brown UniversityProvidence, RI, USA

**Keywords:** opaque, *C. albicans*, zebrafish model, temperature, virulence

## Abstract

Phenotypic diversity is critical to the lifestyles of many microbial species, enabling rapid responses to changes in environmental conditions. In the human fungal pathogen *Candida albicans*, cells exhibit heritable switching between two phenotypic states, white and opaque, which yield differences in mating, filamentous growth, and interactions with immune cells *in vitro*. Here, we address the *in vivo* virulence properties of the two cell states in a zebrafish model of infection. Multiple attributes were compared including the stability of phenotypic states, filamentation, virulence, dissemination, and phagocytosis by immune cells, and phenotypes equated across three different host temperatures. Importantly, we found that both white and opaque cells could establish a lethal systemic infection. The relative virulence of the two cell types was temperature dependent; virulence was similar at 25°C, but at higher temperatures (30 and 33°C) white cells were significantly more virulent than opaque cells. Despite the difference in virulence, fungal burden, and dissemination were similar between cells in the two states. Additionally, both white and opaque cells exhibited robust filamentation during infection and blocking filamentation resulted in decreased virulence, establishing that this program is critical for pathogenesis in both cell states. Interactions between *C. albicans* cells and immune cells differed between white and opaque states. Macrophages and neutrophils preferentially phagocytosed white cells over opaque cells *in vitro*, and neutrophils showed preferential phagocytosis of white cells *in vivo*. Together, these studies distinguish the properties of white and opaque cells in a vertebrate host, and establish that the two cell types demonstrate both important similarities and key differences during infection.

## Introduction

*Candida albicans* is a commensal yeast found colonizing the mouth, gastrointestinal, and reproductive tracts of approximately 70% of healthy individuals (Ruhnke and Maschmeyer, 2002). However, in immunocompromised individuals, *C. albicans* can invade organs and cause serious, life-threatening systemic infections (Garcia-Vidal et al., 2013). The ability of *C. albicans* to exist as both a harmless commensal and as a deadly pathogen is due, at least in part, to its ability to undergo rapid and reversible phenotypic changes (Zordan et al., 2006; Alby and Bennett, 2009; Lohse and Johnson, 2009; Sudbery, 2011; Pande et al., 2013; Tao et al., 2014). In particular, *C. albicans* can switch between yeast and filamentous forms, and this transition is closely associated with the ability to cause disease in the host (Lo et al., 1997; Saville et al., 2003; Zheng et al., 2004).

*C. albicans* can also undergo phenotypic switching between different cellular states, as exemplified by heritable switching between “white” and “opaque” forms (Slutsky et al., 1987). *C. albicans* white and opaque cells have distinctive cellular appearances; white cells are spherical and give rise to bright, dome-shaped colonies, whereas opaque cells are elongated and give rise to darker, flatter colonies (Slutsky et al., 1987). White and opaque cells also differ in other attributes including their gene expression profiles, their ability to mate, the conditions in which they undergo filamentation, their interactions with immune cells, and their virulence in a mouse tail vein model of systemic candidiasis (Kvaal et al., 1997; Lan et al., 2002; Miller and Johnson, 2002; Lohse and Johnson, 2008; Tuch et al., 2010; Si et al., 2013).

The regulation of the epigenetic white-opaque switch has been examined in detail and involves distinct transcriptional networks in the two cell types. The master regulator of the opaque state is Wor1, a transcription factor whose expression is necessary and sufficient for opaque cell formation (Huang et al., 2006; Srikantha et al., 2006; Zordan et al., 2006, 2007). Thus, cells that overexpress Wor1 are locked in the opaque state *in vitro*, whereas cells that lack Wor1 are locked in the white state. Wor1 also acts in concert with at least five other transcription factors to regulate the white-opaque switch via a network of positive and negative feedback loops (Zordan et al., 2007; Wang et al., 2011; Hernday et al., 2013). In addition to transcriptional control, the white-opaque transition is regulated by chromatin modifications including acetylation of histone H3K56 and deposition of histone H2A.Z (Hnisz et al., 2009; Stevenson and Liu, 2011, 2013). Switching is also regulated by the mating type locus; opaque formation occurs predominantly in **a** or α cells (Miller and Johnson, 2002), although switching has also been documented in some **a**/α isolates (Xie et al., 2013).

Despite a detailed understanding of the mechanism of white-opaque switching, there is limited information about how white and opaque cell types differ in their interactions with the host. *In vitro* studies suggest that opaque cells are more susceptible to killing by neutrophils than white cells, and also stimulate greater superoxide production (Kolotila and Diamond, 1990), whereas only white cells release a chemoattractant for neutrophils (Geiger et al., 2004). Furthermore, white cells are more efficiently phagocytosed by macrophages and neutrophils than opaque cells *in vitro* (Lohse and Johnson, 2008; Sasse et al., 2013), indicating that opaque cells may be less visible to immune components. In contrast, both cell types are phagocytosed with equal efficiency by dendritic cells (Sasse et al., 2013), while only white cells secrete E, E-farnesol, a stimulator of macrophage chemokinesis (Hargarten et al., 2015).

In addition to differential interactions with immune cells, white and opaque cell types exhibit different niche specificities during infection of a mammalian host. Opaque cells preferentially colonize the skin (Lachke et al., 2003), whereas white cells are more virulent in a murine model of systemic infection (Kvaal et al., 1997, 1999). It was originally thought that opaque cells could not stably exist inside the host, as opaque cells are unstable at 37°C *in vitro*, rapidly switching back to the white form (Slutsky et al., 1987). However, it was subsequently shown that opaque cells could be maintained at elevated temperatures in certain environmental conditions, including anaerobiasis, 5% CO_2_, and the presence of N-acetylglucosamine (Dumitru et al., 2007; Ramírez-Zavala et al., 2008; Huang et al., 2009, 2010). Furthermore, studies have identified conditions that support the propagation of opaque cells within the host, including evidence that the gastrointestinal tract can promote white-to-opaque switching in certain isolates (Ramírez-Zavala et al., 2008). Despite these studies, questions remain as to the precise roles of white and opaque cells during infection, as well as the traits of the two cell types that give rise to different properties *in vivo*.

Zebrafish (*Danio rerio*) have been developed as an alternative model system for understanding interactions between microbial pathogens and the vertebrate host (Torraca et al., 2014). Experimental models using egg, larval, and adult zebrafish have been developed for *C. albicans*, and have examined both mucosal and disseminated disease (Chao et al., 2010; Brothers et al., 2011; Brothers and Wheeler, 2012; Gratacap et al., 2014; Chen et al., 2015). Zebrafish can be maintained at a wide range of temperatures (from 23 to 33°C), which allows for temperature-dependent phenomena to be investigated. Additionally, the innate immune system of the zebrafish, which is comprised of macrophages, neutrophils, complement, and Toll-like receptors (TLRs) (Seeger et al., 1996; Herbomel et al., 1999; Jault et al., 2004; Meijer et al., 2004; Le Guyader et al., 2008), has a high degree of conservation with mammals, allowing for greater understanding of host-pathogen interactions (Meeker and Trede, 2008; Novoa and Figueras, 2012; van der Vaart et al., 2012). Adaptive immunity develops approximately 2–3 weeks post-fertilization (Willett et al., 1999; Danilova and Steiner, 2002; Lam et al., 2004), thus experiments with zebrafish larvae specifically assess interactions with the innate immune system. The transparency of zebrafish also permits direct, non-invasive visualization of both host and microbial cells at early life stages (Meeker and Trede, 2008; Knox et al., 2014). Previous experiments infected zebrafish with *C. albicans* white cells and established that virulence is dependent on the yeast-hyphal switch, and also showed that host resistance requires NADPH oxidase activity, indicating parallels with disseminated candidiasis in mammalian models of infection (Brothers et al., 2011; Gratacap and Wheeler, 2014).

In this study, we compare and contrast the ability of white and opaque forms of *C. albicans* to infect zebrafish larvae. These experiments compare infection, filamentation, dissemination, phagocytosis, and virulence by both cellular states, and contrast these properties over a range of host temperatures. Interestingly, pathogenicity is temperature dependent, with white cells being more virulent than opaque cells at 30 and 33°C, but not at 25°C. Both white and opaque cells formed filaments *in vivo*, which is the first demonstration that opaque cells can undergo filamentation inside a vertebrate host. Analysis of mutants unable to undergo filamentation show that this program contributes to virulence by both white and opaque cells. Furthermore, we examined phagocytosis of white/opaque cells *in vitro* and *in vivo*, and reveal significant differences in their interactions with host immune cells. We discuss these differences in light of the role of pathogen-phagocyte interactions in promoting susceptibility to lethal infection by *C. albicans*.

## Materials and methods

### *C. albicans* strains and media

*C. albicans* strains are listed in Table 1. Media was prepared as described (Guthrie, 1991). Yeast extract peptone dextrose (YPD) plates containing 100–200 μg/ml nourseothricin (NAT) were used for selection of strains that were resistant to nourseothricin (Reu et al., 2004). All strains were stored as frozen stocks with 25% glycerol at −80°C.

**Table 1:** **Strain list**.

**Name**	**Genotype**	**MTL**	**References**
RBY717	MTL**a**/MTLa^*^ (white)	**a**/**a**	Bennett and Johnson, 2006
RBY731	MTL**a**/MTLa^*^ (opaque)	**a**/**a**	Bennett and Johnson, 2006
CAY4975	MTL**a**/MTL**a** pEno1-dTomato::SAT1^*^ (white)	**a**/**a**	This study
CAY4986	MTL**a**/MTL**a** pAct1-WOR1::HYG pEno1-dTomato::SAT1^*^ (opaque)	**a**/**a**	This study
CAY5202	MTL**a**/MTL**a** pEno1-dTomato::SAT1^*^ (opaque)	**a**/**a**	This study
CAY715	L26 *MTL**a**/MTL**a*** (white)	**a**/**a**	Lockhart et al., 1996
CAY6548	L26 *MTL**a**/MTL**a** pACT1-WOR1::SAT1* (opaque)	**a**/**a**	This study
CAY713	12C *MTL**a**/MTL**a*** (white)	**a**/**a**	Lockhart et al., 1996
CAY6814	12C *MTL**a**/MTL**a** pAct1-WOR1:SAT1* (opaque)	**a**/**a**	This study
CAY716	P37005 *MTL**a**/MTL**a*** (white)	**a**/**a**	Lockhart et al., 1996
CAY1477	P37005 *MTL**a**/MTL**a*** (opaque)	**a**/**a**	Lin et al., 2011
CAY6597	*MTL**a**/MTLα Δ/Δcph1/cph1 Δ/Δefg1/efg1 pEno1-dTomato::SAT1* (white)	**a**/α	This study
CAY3292	MTL**a**/MTLαΔ::FRT efg1::LEU2/efg1::HIS1 arg4::ARG4/arg4 leu2/leu2 his1/his1 pAct1-WOR1::SAT1^*^ (opaque)	**a**/Δα	Si et al., 2013
CAY4384	MTL**a**/MTLαΔ::FRT efg1::LEU2/efg1::HIS1/EFG1::HYG leu2/leu2 his1/his1 arg4::ARG2/arg4 pAct1-WOR1::SAT1^*^ (opaque)	**a**/Δα	Si et al., 2013
CAY7071	*MTL**a**/MTLαΔ::FRT Δ/Δcph1/cph1 Δ/Δefg1/efg1 pAct1-WOR1::FRT pEno1-dTomato::SAT1* (opaque)	**a**/Δα	This study
CAY1579	*MTL**a**/MTLαΔ::FRT Δ/Δcph1/cph1 Δ/Δefg1/efg1 pAct1-WOR1::SAT1* (opaque)	**a**/Δα	This study

* *strains also contain the genotype ura3::imm434/URA3 iro1::imm434/IRO1*.

To create a fluorescently labeled white strain for fish injections (RBY4975), a construct containing the P_*ENO*1_ promoter, a codon-optimized dTomato gene, the T_*TEF*_ terminator, and a NAT^r^ marker that has been previously described (Brothers et al., 2013; Gratacap et al., 2013) was transformed into strain RBY717. Transformants were selected by plating on nourseothricin and screened for correct integration at the *ENO1* locus by PCR and by fluorescence. To switch RBY4975 to the opaque state, the strain was grown on Lee's + N-acetylglucosamine at 37°C with 5% CO_2_ and the resulting opaque strain named CAY5202. To promote the opaque state, a *pACT1-WOR1* plasmid (Si et al., 2013) was transformed into RBY4975 to create RBY4986. To construct strain CAY7071 (Δ*/*Δ*cph1/cph1* Δ*/*Δ*efg1/efg1 pACT1-WOR1::FRT pEno1-dTomato::SAT1*), CAY6597 was first transformed with pRB102 (a construct to delete *MTL*α) that was linearized with ApaI and SacI, as described (Alby and Bennett, 2009). Transformants were selected by growth on nourseothricin and verified by PCR. The *SAT1* flipper cassette was removed as described (Reu et al., 2004), and the strain transformed with *pACT1-WOR1*; transformants were selected on nourseothricin. Colonies were verified by microscopy to ensure they were opaque, and by PCR to verify correct integration of the construct. The *SAT1* flipper cassette was removed and a construct containing the P_*ENO*1_ promoter, a codon-optimized dTomato gene, the T_*TEF*_ terminator, and a NAT^r^ marker (Brothers et al., 2013; Gratacap et al., 2013) transformed into the strain. Transformants were selected by plating on nourseothricin and screened for correct integration at the *ENO1* locus by PCR and by fluorescence.

### Preparation of *C. albicans* cells for microinjection

Zebrafish experiments were performed both at Brown University and at the University of Maine. For experiments performed at Brown University, *C. albicans* strains were grown overnight in SCD medium at 22°C, washed three times with phosphate-buffered saline (PBS), adjusted to a concentration of 1 × 10^9^ cells/ml, and stained with AlexaFluor-488 or 568 carboxylic acid, succinimidyl ester (Invitrogen, Grand Island, NY) as previously described (Wheeler et al., 2008). Briefly, 100 μl of *C. albicans* cells were stained with 1.5 μl of 10 mg/ml AlexaFluor-488 or 568 carboxylic acid, succinimidyl ester for 30 min in the dark. *C. albicans* cells were washed three times with PBS and resuspended in PBS containing 5% polyvinylpyrrolidone (PVP) (Sigma-Aldrich, St. Louis, MO) to a concentration of 5 × 10^8^ cells/ml. Low concentration PVP is commonly used to ensure a consistent small-volume microinjection dose (Detrich et al., 2010).

For experiments performed at the University of Maine, *C. albicans* strains CAY4975 and CAY4986 were grown at 21°C on SCD medium for 48 h, washed twice with PBS, stained with 1.5 μl of 10 mg/ml AlexaFluor-647 carboxylic acid, succinimidyl ester (Invitrogen, Grand Island, NY) in order to distinguish inoculum and newly divided *C. albicans* cells, and resuspended in 5% w/v PVP (Sigma-Aldrich, St. Louis, MO) to a final concentration of 5 × 10^7^ cells/ml.

### Zebrafish care and maintenance

At Brown University, wild type zebrafish were obtained from Carolina Biological Supply and housed in multiple recirculating 10 and 20 gallon tanks or in the Aquatic Habitats system (Apopka, FL). Zebrafish care protocols and experiments were performed in accordance with NIH and Brown University guidelines under Brown University Institutional Animal Care and Use Committee (IACUC) protocols 1210031 (Richard Bennett) and 1207022 (Robbert Creton). Water temperature in tanks was maintained at 28°C and fish were fed a combination of Gemma fish food, frozen brine shrimp, and freeze-dried bloodworms. Zebrafish were kept in mixed male and female populations under a 14 h light/10 h dark cycle. Embryos were collected from the tanks at the beginning of the light cycle (“dawn”) and grown at a density of 50/dish in 10-cm petri dishes containing 60 ml egg water (deionized water with 60 mg/L Instant Ocean salts [United Pet Group Inc. Cincinnati, OH]) and kept at 28°C. To prevent microbial growth, egg water was supplemented with 0.00003% methylene blue (Fisher, Fair Lawn, NJ) for the first 24 h. Larval egg water was changed daily.

At the University of Maine, zebrafish were housed at the Zebrafish Facility in a recirculating system with a flow rate of 167 liters/min (Aquatic Habitats, Apopka, FL) on a 10/14-h dark/light cycle, respectively. Water was maintained at 28°C and mixed populations of males and females were kept in 3-liter tanks. Zebrafish care protocols and experiments were performed in accordance with NIH guidelines under University of Maine IACUC protocol A2012-11-03. Larvae were grown at a density of 150/dish in 150 mm petri dishes containing 150 ml of E3 (5 mM sodium chloride, 0.174 mM potassium chloride, 0.33 mM calcium chloride, 0.332 mM magnesium sulfate, 2 mM HEPES in Nanopure water, *pH* = 7). For transgenic experiments the fish strain used was Tg(mpeg1:Gal4 UAS-nfsB-mcherry mpx:EGFP) [72, 73] in which neutrophils express enhanced green fluorescent protein and macrophages express mCherry red fluorescent protein. E3 media was supplemented with 0.3 mg/L methylene blue for the first 6 h to prevent microbial growth. Larvae were transferred to E3 containing 0.02 mg/ml of 1-phenyl-2-thiourea (PTU) (Sigma-Aldrich, St. Louis, MO) to prevent pigmentation and kept at 33°C.

### Zebrafish hindbrain microinjections

At Brown University, zebrafish in the Prim25 stage (~36 h post-fertilization) were manually dechorionated and anesthetized in Tris-buffered tricaine methane sulfonate (tricaine, 0.04%) (Western Chemical Inc., Ferndale, WA). Glass capillary tubes (World Precision Instruments, Inc. Sarasota, FL) were pulled and loaded with 10 μl of PBS or a *C. albicans* strain at a concentration of 5 × 10^8^ cells/ml using MicroFil syringe needles (World Precision Instruments, Inc., Sarasota, FL). Needles were clipped to achieve a bolus size of 5–10 nl, which was measured using a 0.01 mm calibration slide. Larvae were aligned on a 2% agarose dish and injected through the otic vesicle into the hindbrain ventricle using a Leitz Wetzlar micromanipulator and microinjection machine. Following injection, larvae were individually screened microscopically in glass bottom 96-well plates (Greiner Bio-One, Monroe, NC) to quantify the number of *C. albicans* injected into the hindbrain using a Zeiss Axio microscope. Larvae were divided into groups based on the inoculum range, kept at 25, 30, or 33°C, and monitored daily for survival. Egg water was changed daily.

At the University of Maine, injections were performed on zebrafish at the Prim25 stage that were staged according to the method of Kimmel et al. (1995). Fish were manually dechorionated, anesthetized in Tris-buffered tricaine methane sulfonate (Tricaine; 200 mg/ml) (Western Chemicals, Inc., Frendale, WA), and ~5 nl of *C. albicans* white or opaque cell suspensions at 5 x 10^7^ CFU/ml in 5% PVP was microinjected through the otic vesicle into the hindbrain ventricle to achieve a dose of 20–50 fungal cells at the site of injection. Within 1 h post-injection, larvae were screened using a Zeiss Axiobserver Z1 microscope equipped with the Vivatome system (Carl Zeiss Microimaging, Thornwood, NJ) for selection of larvae containing 20–50 fungal cells in the hindbrain ventricle. Larvae were subsequently incubated at 33°C in E3 containing PTU.

### Fluorescence microscopy

For experiments done at Brown University, DIC and fluorescent images were collected with a Zeiss Inverted Microscope (Axio Observer. Z1) fitted with an AxioCam HR. Images were processed with AxioVision Rel. 4.8 (Zeiss, Germany) and ImageJ. Confocal images were collected with a LSM 510 Meta at the Leduc BioImaging Facility at Brown University and processed using Zen software, ImageJ, and Adobe Photoshop.

For experiments performed at the University of Maine, at 4 h post-injection (HPI) larvae were anesthetized in Tricaine and immobilized in 0.5% low-melting-point agarose (Lonza) in E3 containing Tricaine in a 96-well glass-bottom imaging dish (Greiner Bio-One, Monroe, NC). Confocal images were acquired using an Olympus IX-81 inverted microscope with an FV-1000 laser scanning confocal system (Olympus). The EGFP and dTomato fluorescent proteins were detected by laser/optical filters with a 40x objective (NA 0.75) for excitation/emission at 488/505–525 nm and 543/560–620 nm, respectively. Images were collected and processed using Fluoview (Olympus) and Photoshop (Adobe Systems, Inc.). Images were manually analyzed with Fluoview to morphologically categorize *Candida*/phagocyte interactions at the site of injection. EGFP-expressing cells were scored as neutrophils, mCherry-expressing cells were scored as macrophages, and non-fluorescent phagocytic cells were scored as macrophages presumed to have a silenced UAS promoter due to mosaic transgenerational silencing. After imaging, larvae were euthanized by Tricaine overdose.

### Switching assays

To measure the stability of opaque (RBY731 or CAY5202) and *WOR1*-overexpressing (CAY4986) cells at different temperatures, a single colony was inoculated into 5 ml SCD medium and grown overnight at 25, 30, or 33°C. Each day, cultures were diluted 1:100 into 5 ml fresh SCD medium. In addition, 100 CFU were plated onto SCD and grown for 7 days at 22°C to determine the number of white and opaque colonies. 5 biological replicates were performed for each strain at each temperature.

### Enumeration of fungal burdens

Individual zebrafish were placed in eppendorf tubes containing 250 μl PBS supplemented with antibiotics [penicillin (500 μg/ml), ampicillin (500 μg/ml), kanamycin (250 μg/ml), doxycycline (125 μg/ml), streptomycin (250 μg/ml), and chloramphenicol (125 μg/ml)]. Fish were homogenized using a sample pestle with a Micro Grinder (Research Products International Corp.). Serial dilutions were made in PBS containing antibiotics and plated onto SCD medium. Plates were incubated at 22°C for 7 days prior to counting.

### Assessment of dissemination

To determine the degree of dissemination, larvae were infected with 21–50 *C. albicans* cells, and kept at 25, 30, or 33°C. At 2 days post-infection, fish were euthanized and heads were separated from the rest of the body/tail. Each segment (head and body/tail) was homogenized in PBS supplemented with antibiotics [penicillin (500 μg/ml), ampicillin (500 μg/ml), kanamycin (250 μg/ml), doxycycline (125 μg/ml), streptomycin (250 μg/ml), and chloramphenicol (125 μg/ml)] using a sample pestle with a Micro Grinder (Research Products International Corp.). Serial dilutions of each body segment were made in PBS containing antibiotics and plated onto SCD plates. Plates were incubated at 25°C for 7 days prior to counting.

### Assessment of filamentation by fish crushing

To assess filamentation, larvae were infected with 21–50 *C. albicans* cells and kept at 25, 30, or 33°C. At the indicated time point post-infection, fish were crushed between a glass slide and coverslip as previously described (Brothers et al., 2011) and screened microscopically for filamentous fungi.

### Growth curves

5 ml overnight *C. albicans* cultures were grown at 25°C in SCD, washed twice in H_2_0 and inoculated into 96-well plates at an OD_600_ of 0.1 in 200 μl of YPD, SCD, or RPMI. Plates were incubated at 25, 30, or 33°C in a Biotek Synergy HT plate reader for 24 h. OD_600_ measurements were made at 15 min intervals. Data was analyzed using a previously published Matlab script (Abbey et al., 2011) and fit to a polynomial curve. Each well was examined microscopically after the assay reached completion and analyzed for cellular phenotype and filamentation. To convert OD_600_ at saturation to cells/ml, cell concentration was determined using a hemocytometer.

### Purification of human PMNs

This study was carried out in accordance with the recommendations of Rhode Island Hospital Institutional Review Board (IRB 1–00000396 and RIH IRB 2 – 00004624 to JR). All subjects gave written informed consent in accordance with the Declaration of Helsinki. Blood was obtained from healthy human volunteers and isolation of PMNs was performed as described (Byrd et al., 2013). Briefly, blood was collected in EDTA-containing Vacutainer tubes (BD Biosciences, San Jose, CA) and used within 5 min of venipuncture. Histopaque-1077 was used for initial cell separation followed by sedimentation through 3% dextran (400–500 kDa). Contaminating erythrocytes were removed by hypotonic lysis, yielding a >95% pure neutrophil preparation with >90% viability by trypan dye exclusion. Neutrophils were suspended in HBSS (without Ca^2+^/Mg^2+^) and placed on ice until use.

### Isolation of bone marrow derived macrophages (BMDM)

BMDM were isolated as previously described (Aubry et al., 2012). Briefly, tibia and femur bones from 6 to 8 week-old C57BL/6J mice were collected in ice cold PBS. Bones were sterilized with 70% ethanol and flushed with a 25-G needle using cold DMEM supplemented with 10% FCS, 10% L929 conditioned medium (LCM), and 1% penicillin-streptomycin. Cells were seeded onto 6-well plates (Nunc) at a concentration of 10^6^ cells per well and incubated at 37°C with 5% CO_2_. After 4 days, complete medium was added and cells were split at a ratio of 1:2. After 8 days, macrophages were fully differentiated.

### Cell lines

*D. melanogaster* S2 cells were cultured at 22–28°C in Schneider's medium (Invitrogen, Carlsbad, California) supplemented with 10% heat inactivated fetal bovine serum (FBS), penicillin, and streptomycin (pen/strep) (0.1 mg/mL, 100 U/mL). *M. musculus* RAW 264.7 cells were cultured in DMEM (Corning, Manassas, VA) supplemented with 10% heat inactivated FBS, 0.002 M L-glutamine, and pen/strep (0.1 mg/mL, 100 U/mL), and grown at 37°C, 5% CO_2_.

### Phagocytosis assays

Phagocytosis assays were performed similarly to those previously described (Lohse and Johnson, 2008), with a few modifications. For assays using S2 cells, cells were seeded in a tissue culture treated 96-well plate at a density of 0.5 × 10^5^ cells/well and grown overnight at 22–28°C. Overnight cultures of *C. albicans* were diluted 1:50 and grown for 3 h at 25°C. These cultures were washed three times with PBS, resuspended in cell culture medium at a concentration of 4 × 10^6^ cells/ml, and 100 μl of fungal cells were co-cultured with S2 cells for 1 h at 28°C. S2 cells and *C. albicans* were then transferred to a Con-A-coated glass bottom 96-well plate (Greiner Bio-One, Monroe, NC) and incubated an additional hour. For phagocytosis assays using RAWs and BMDMs, cells were seeded in glass bottom 96-well plates (Greiner Bio-One) at a density of 1 × 10^5^ cells/well and grown overnight at 37°C with 5% CO_2_. The next day, supernatant was removed, cells were washed 1–2 times with PBS, and 150 μl fresh cell culture media was added. Overnight cultures of *C. albicans* were diluted 1:50 and grown for 3 h at 25°C. These cultures were washed three times with PBS, resuspended in cell culture media at a concentration of 4 × 10^6^ cells/ml, and 100 μl of fungal cells were co-cultured with RAWs or BMDMs for 1 h at 37°C, 5% CO_2_. For phagocytosis assays using human neutrophils, glass bottom 96-well plates (Greiner Bio-One) were coated with 10 μg/ml human fibronectin in TBS, pH 9, overnight at 4°C. The following day, wells were washed twice with PBS and human neutrophils were seeded onto each well at a density of 2 × 10^5^ cells/well in L15 media containing 10^−9^ M fMLP and 2 mM Mn^2+^. Overnight cultures of *C. albicans* diluted 1:50 and grown for 3 h at 25°C. These cultures were washed three times with PBS, resuspended in cell culture medium at a concentration of 4 × 10^6^ cells/ml, and 100 μl of fungal cells were added to each well and co-incubated with PMNs for 30 min at 37°C, 5% CO_2_.

After co-incubation of immune cells with *Candida*, media was removed and wells were air dried for 2 min. Cells were fixed with 1% formaldehyde for 5 min, washed, and blocked with 5% FBS in PBS for 1 h. During this time, a primary anti-*C. albicans* antibody (Biodesign International, Saco, ME) was pre-blocked in 5% FBS in PBS at a 1:1000. Blocking solution was removed and the primary antibody was added and incubated overnight at 4°C. Wells were washed twice with PBS for 5 min. A secondary antibody (FITC-conjugated donkey-anti-rabbit IgG (H+L)) (Jackson Laboratories, Bar Harbor, ME) was then added (1:1000 in 5% FBS in PBS) and incubated for 2 h at 25°C. Wells were washed twice with PBS for 5 min each and Hoechst stain (Invitrogen, Eugene, Oregon) was added to each well (1:2000 dilution of 10 mg/ml in PBS) for 30 min. Hoechst stain was removed and 50 μl of fluoromount (Southern Biotech, Birmingham, AL) added to each well.

Wells were examined microscopically and the percent phagocytosis and phagocytic index (PI) were determined as described (Lohse and Johnson, 2008). Each experimental condition was tested in duplicate or triplicate wells and percent phagocytosis and phagocytic index were calculated for at least 2 sets of 100 macrophage cells from each well. Each experiment was performed at least 3 times.

### Statistics

Differences in outcomes between strains were evaluated using Student's *t*-tests for two group comparisons, and one-way and two-factor repeated measures anova appropriately for more complex designs. In the presence of significant effects, pairwise comparisons were made using Tukey's HSD multiple comparisons procedure in the case of the one-way anova, and Bonferroni adjusted Fisher's LSD tests in the case of the two-factor repeated measures anova. Differences in survival times were evaluated using Kaplan Meier Product Moment Survival Analysis with Log Rank tests to evaluate significance. Multivariate survival analysis was performed using Cox Proportionate Hazards models. The proportionate hazards assumption was tested by visual inspection of cumulative survival plots. Statistical significance was defined as effects with *p*-values less than or equal to 0.05. ^*^p < 0.05, ^**^p < 0.01, ^***^p < 0.001, and ^****^p < 0.0001. Analyses were performed using GraphPad Prism and SPSS version 15.

## Results

### *C. albicans* white and opaque cells establish a systemic infection in the zebrafish model

To compare the properties of *C. albicans* white and opaque cells we used the zebrafish hindbrain model of infection (Brothers et al., 2011; Brothers and Wheeler, 2012). Wild type larvae were injected with AlexaFluor-488 labeled *C. albicans* cells expressing a dTomato reporter (see Table 1). The AlexaFluor-488 signal was used to identify *C. albicans* cells immediately after infection and dTomato expression used for subsequent identification of *C. albicans* cells during long-term infections. Unless stated otherwise, experiments were performed with white and opaque **a**/**a** cells derived from SC5314, the standard laboratory strain of *C. albicans*.

**Figure 1:**
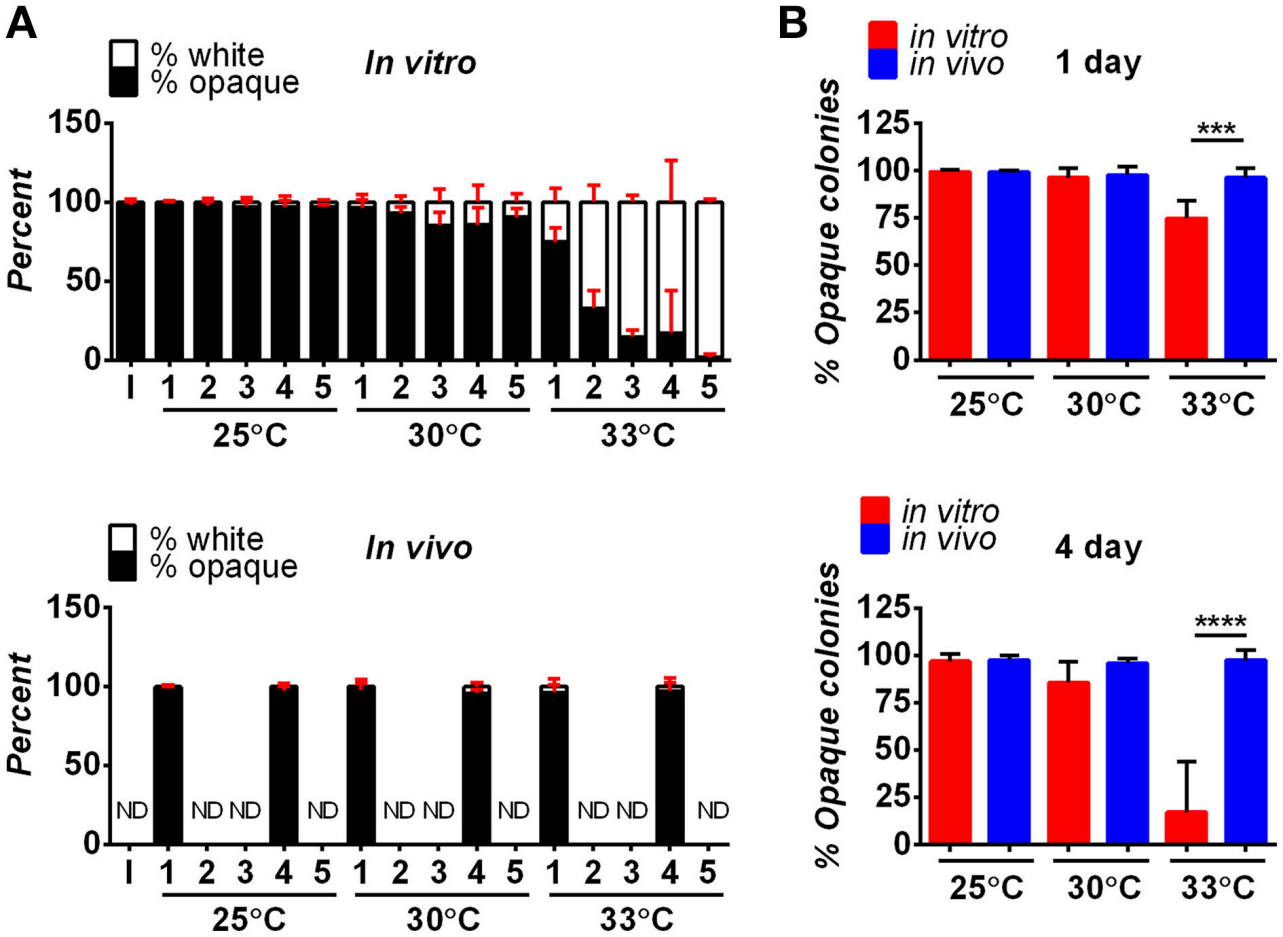
**Stability of opaque cells ***in vitro*** and ***in vivo***. (A)** Opaque cells (CAY5202) were cultured *in vitro* (top panel) or in zebrafish larvae *in vivo* (bottom panel) at the indicated temperatures. For the *in vitro* experiment, cultures were grown in SCD medium at the indicated temperature, and diluted back daily into fresh SCD medium. Each day, ~100 cells were plated onto SCD and the fraction of white and opaque colonies was determined. Shown are the mean percentages of white and opaque colonies of 6 biological replicates at each time point for each temperature. For *in vivo* experiments, larvae were infected with 5–50 CFU of one of six different biological replicates of CAY5202 and housed at 25, 30, or 33°C. At 1 and 4 days post-infection, individual fish were homogenized and serial dilutions were plated onto SCD to determine the fraction of white and opaque cells present. Shown are the mean percentages of white and opaque colonies at each time point for each temperature ± SD. ND, not determined; I, initial inoculum. **(B)** Comparison of the stability of opaque cells *in vitro* and *in vivo* at 1 and 4 days. Shown are the mean percentages of opaque cells ± SD. Data are a compilation of two independent experiments using 6 biological replicates at each time point for each temperature. Statistically significant differences were determined using a Student's *t*-test. ^***^p < 0.001; ^****^p < 0.0001.

The stability of white and opaque cells was compared *in vitro* and *in vivo* at three different temperatures: 25, 30, and 33°C (the latter being the upper limit for zebrafish experiments). The stability of opaque cells has previously been shown to be temperature sensitive; culture of opaque cells at 34°C (or higher) led to switching *en masse* to the white state (Slutsky et al., 1987; Rikkerink et al., 1988). We also observed instability of opaque cells at elevated temperatures *in vitro*. Opaque cells were stably maintained during culture in SCD medium at 25 or 30°C (Figures 1A,B). At 33°C, however, high levels of opaque-to-white switching were observed, so that after 2 days ~67% of cells had switched to the white state, and by 5 days ~98% of cells were in the white state (Figures 1A,B). In contrast to *in vitro* experiments, opaque cells were stable *in vivo* at all three temperatures. Thus, < 5% of opaque cells had switched to the white state after 4 days of infection at 25, 30, or 33°C (Figures 1A,B). Furthermore, the majority of opaque cells were stable even out to 7 days post-inoculation, regardless of temperature (Figure S1A). These results show that opaque cells are significantly more stable in the zebrafish host than during standard *in vitro* culture conditions. Thus, *in vivo* conditions are permissive for stable propagation of opaque cells during disseminated candidiasis.

To help ensure that cells stably maintained the opaque state, strains were constructed in which *WOR1*, the master regulator of the opaque state (Huang et al., 2006; Srikantha et al., 2006; Zordan et al., 2006, 2007), was constitutively expressed. Compared to control opaque cells, *WOR1*-overexpressing (*WOR1* OE) cells displayed significantly increased stability after *in vitro* passaging at 30 or 33°C (Figure S1B). Furthermore, when *WOR1* OE cells were used in zebrafish experiments, 100% of recovered cells retained the opaque state after 7 days infection at 25, 30, or 33°C (Table S1). These results again indicate that opaque cells are more stably maintained *in vivo* than *in vitro*, especially at 33°C (see Table S1, Figures 1A,B). Due to their increased stability, *WOR1* OE strains were used for infections with opaque cells, unless stated otherwise.

**Figure 2:**
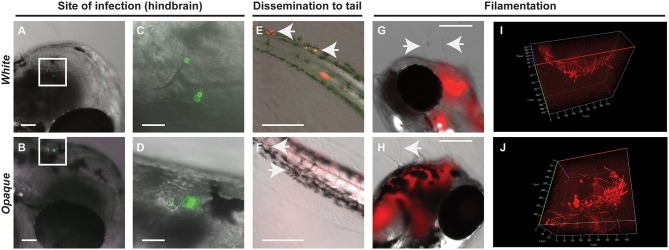
**Both white and opaque phenotypic states of ***C. albicans*** infect, disseminate, and are induced to undergo filamentation *in vivo***. **(A)** Hindbrain of wild type zebrafish in the infected with 8 *C. albicans* white cells (CAY4975) pre-stained with AlexaFluor-488 (see Methods) at 30 min post-injection. Magnification, 10 ×; scale bar, 50 μm. White box indicates *C. albicans* cells in the hindbrain and this area is shown at higher magnification in **(C)**. **(B)** Hindbrain of wild type zebrafish infected with 12 *C. albicans* opaque cells (CAY4986) pre-stained with AlexaFluor-488 (see Methods) at 30 min post-infection. Magnification, 10 ×; scale bar, 50 μm. White box indicates *C. albicans* cells present in the hindbrain shown at higher magnification in **(D)**. **(C)** Higher magnification of white box in **(A)**. Magnification, 40 ×; scale bar, 20 μm. **(D)** Higher magnification of white box in **(B)**. Magnification, 40 ×; scale bar, 20 μm. **(E)** Wild type zebrafish were infected with >200 *C. albicans* white cells expressing a dTomato reporter (CAY4975) and kept at 25°C. Shown are *C. albicans* cells that have disseminated to the tail (see arrows) at 2 days post-infection. Magnification, 10 ×. Scale bar, 200 μm. **(F)** Wild type zebrafish were infected with 50 opaque *C. albicans* cells expressing dTomato (CAY4986) and kept at 25°C. Shown are *C. albicans* cells that have disseminated to the tail (see arrows) at 1 day post-infection. Magnification, 10 ×; scale bar, 200 μm. **(G)** 10 × image of wild type zebrafish infected with 25 *C. albicans* white cells expressing a dTomato reporter (CAY4975) at 9 days post-infection. Arrows indicate fungal filaments penetrating out of the fish's head. Scale bar, 200 μm. **(H)** 10 × image of wild type zebrafish infected with 100 *C. albicans* opaque cells expressing a dTomato reporter (CAY4986) and stained with AlexaFluor-488 at 5 days post-infection. Arrow indicates filamentation out of the fish's head. Scale bar, 200 μm. **(I)** Confocal microscopy of zebrafish infected with *C. albicans* white cells (CAY4975) expressing a dTomato reporter at 1 day post-infection and exhibiting filamentation. Magnification, 20 ×. **(J)** Confocal microscopy of zebrafish infected with *C. albicans* opaque cells expressing a dTomato reporter (CAY4986) at 1 day post-infection and exhibiting filamentation. Magnification, 20 ×.

Following inoculation, both white and opaque forms of *C. albicans* were detected in the hindbrain of the fish using the AlexaFluor-488 signal (Figures 2A–D). By day one post-infection, white and opaque cells were found disseminating to the tail of the fish by monitoring expression of the dTomato reporter (Figures 2E,F). Thus, both phenotypic forms are able to establish a systemic infection in the zebrafish model.

### Both white and opaque phenotypic states undergo filamentation *in vivo*

*C. albicans* white and opaque cells undergo filamentation *in vitro* in response to distinct environmental signals. White cells filament in response to cues such as serum and high temperature (37°C), whereas opaque cells show optimal filamentation at ambient temperatures (25°C) in low-phosphate or sorbitol media (Si et al., 2013). Filamentation of white cells has also been observed during oral and systemic infections of a mammalian host (Saville et al., 2003; Sudbery, 2011; Staab et al., 2013), and in the zebrafish model of infection (Brothers et al., 2011), whereas opaque cell filamentation has not been reported *in vivo*.

We compared the ability of *C. albicans* white and opaque cells to undergo filamentation in zebrafish housed at 25, 30, and 33°C. Interestingly, both white and opaque cells formed filaments that penetrated out of the head of some zebrafish larvae at 1-day post-infection (Figures 2G,H). White and opaque forms of *C. albicans* were also both observed to display networks of filaments within the hindbrain of the fish (Figures 2I,J and Movies S1, S2). To establish that filaments observed *in vivo* involved opaque cells, fish were homogenized and serial dilutions of homogenates were plated for single colonies. The overwhelming majority of the plated colonies (~98% for the fish shown in Figure 2J) were opaque colonies, confirming that opaque cells, as well as white cells, undergo filamentation in a vertebrate model of infection.

### Differences in virulence between *C. albicans* white and opaque cells are temperature dependent

White cells were more virulent than opaque cells when compared in a murine model of systemic infection (Kvaal et al., 1997). To assess the virulence of these cell types in the zebrafish model, larvae were infected with white or opaque cells, inoculums determined by microscopy, and infected fish separated into inoculum ranges (e.g., 10–20, 21–50, and 51–100). Experiments were performed at 25, 30, and 33°C (see Methods).

**Figure 3:**
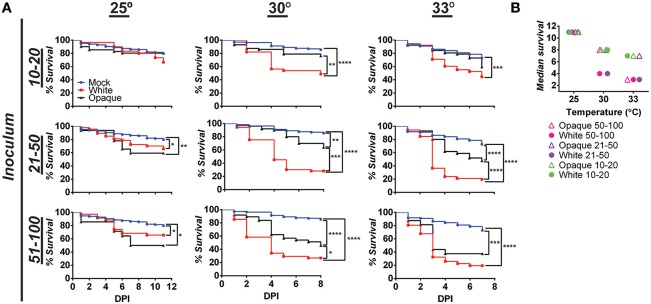
**White cells are more virulent than opaque cells at higher temperatures. (A)** Wild type zebrafish larvae were infected with *C. albicans* white cells (CAY4975), opaque cells (CAY4986), or mock infected. Embryos were kept at the indicated temperature after infection and monitored daily for survival. Data are a compilation of 11, 8, and 7 independent experiments for 25, 30, and 33°C, respectively. *N* = 14–235 fish/group for each inoculation interval. Statistically significant differences were determined using a Log rank (Mantel-Cox) test. ^*^p < 0.05; ^**^p < 0.01; ^***^p < 0.001; ^****^p < 0.0001. **(B)** Median survival (time to 50% survival or endpoint of experiment) was compared for fish infected with the indicated inoculums of *C. albicans* white or opaque cells housed at 25, 30, and 33°C.

When infected fish were kept at 25°C, little mortality was observed at low inoculums (10–20 cells) regardless of cell state (Figure 3A). At higher inoculums (21–50 or 51–100 cells) significant mortality was observed for cells in both phenotypic states vs. mock-treated controls (Figure 3A). Infection with opaque cells showed higher rates of killing than white cells at 25°C, although the difference was not significant. When infections were performed at 30 or 33°C, white cells were significantly more virulent than opaque cells over a range of inocula (Figure 3A). For example, using an inoculum of 21–50 cells at 30°C, white cells resulted in death of 50% of the larvae by day 4, whereas opaque cells did not cause 50% lethality even after 8 days of infection (Figure 3A). These results establish that *C. albicans* white cells are more virulent than opaque cells at elevated temperatures (30 and 33°C), whereas the virulence of the two cell types is similar at ambient temperature (25°C).

We also compared the relative virulence of *C. albicans* cells across the three temperatures and observed that the virulence of both phenotypic states increased with increasing temperature. Thus, the median survival time with an inoculation of 21–50 opaque cells was 11 days at 25°C, 8 days at 30°C, and 7 days at 33°C (Figure 3B). However, in the case of white cells, this trend was even more marked; inoculation with the same number of white cells resulted in a median survival time of 11 days at 25°C, 4 days at 30°C, and 3 days at 33°C. These experiments establish that *C. albicans* virulence increases with temperature, and that this trend is sharper for white cells than for opaque cells.

### Differences in fungal burden and dissemination do not account for virulence differences between white and opaque cells

**Figure 4:**
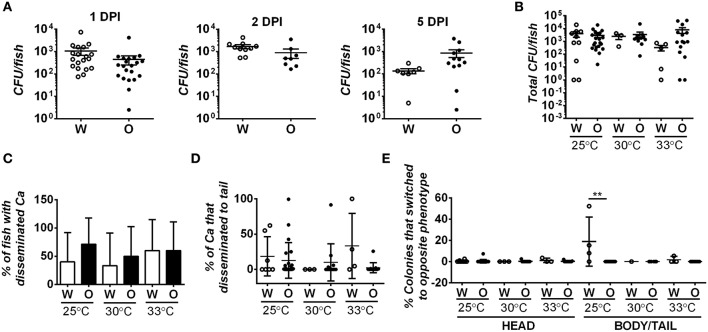
**Fungal burden and degree of dissemination is similar in zebrafish infected with white or opaque cells**. For these experiments zebrafish were injected with 21–50 *C. albicans* white cells (W) or opaque cells (O) (strains CAY4975 and CAY4986, respectively). Data are a compilation of nine individual experiments. **(A)** Fish were housed at 30°C and homogenized at 1, 2, or 5 days post-infection (DPI). Fungal burdens were determined by plating for colony forming units (CFUs). Each dot represents an individual fish and shown are the mean CFUs ± SEM. A Student's *t*-test was performed and no significant differences were present between white- and opaque-infected fish. **(B)** White- and opaque-infected zebrafish were housed at 25, 30, or 33°C. At 2 days post-infection fish were euthanized and heads were dissected from bodies/tails. Serial dilutions of each segment were separately plated for CFUs. Plotted are the mean CFU per fish (head and body/tail segments combined) ± SEM. **(C)** To determine the degree of *C. albicans* (*Ca*) dissemination, white- and opaque-infected zebrafish were euthanized and fish dissected into two segments (head and body/tail). Serial dilutions of both segments were plated for CFUs, and if cells were present in the body/tail then fish were scored as exhibiting dissemination. Error bars represent SD. **(D)** White- and opaque-infected zebrafish were analyzed at 2 days post-infection at each of the three indicated temperatures. Fish were euthanized and heads were dissected from bodies/tails. Serial dilutions of these segments were plated for CFUs and the percentage of *C. albicans* cells that had disseminated into the body/tail compared to that present in the head segment. Plotted are the mean percentages of disseminated *C. albicans* cells ± SD. **(E)** Two days post-infection fish were euthanized and heads were dissected from bodies/tails. Serial dilutions of each segment were plated to determine the phenotypic state of each cell. Those that had switched from the parental white (W) or opaque (O) state to the opposite phenotypic state are shown for both head and body/tail segments. Data is the mean ± SD. ^**^p < 0.01.

Since differences in fungal burdens could contribute to differences in virulence, we assessed fungal burdens in fish infected with 21–50 white or opaque cells and housed at 30°C following inoculation. Fish were homogenized at different time points post-inoculation and the number of colony-forming units (CFUs) per fish was determined. At 1, 2, and 5 days post-infection, we observed similar fungal burdens with both white and opaque cells (Figure 4A). For example, at 1 or 2 days post-infection live fish contained ~2000 CFUs, and this declined by day 5 to an average of less than 900 CFUs per fish (Figure 4A). *C. albicans* CFU levels were also seen to decrease over the course of a zebrafish infection by Brothers et al. (2011). We next assessed fungal CFUs in fish housed at different temperatures. At 2 days post-infection, fungal burdens were similar between white- and opaque-infected fish whether experiments were performed at 25, 30, or 33°C (Figure 4B). These results indicate that differences in virulence between white and opaque cells are not simply due to differences in fungal burdens by the two cell types.

Given that white and opaque cells disseminate from the hindbrain in the larval infection model, we assessed whether differences in dissemination could contribute to differences in virulence. Fish were infected with 21–50 *C. albicans* cells and at 2 days post-infection fish were euthanized and their heads removed from the rest of the body/tail. The head and body/tail segments were homogenized and plated for CFUs and for colony morphology to determine cell state. Approximately half of the fish contained *C. albicans* cells in the body/tail segments, indicative of dissemination (Figure 4C). However, no difference was seen in the degree of dissemination between white and opaque cells in experiments at 25, 30, or 33°C. We also assessed the fraction of *C. albicans* cells that disseminated to the body/tail and, while it was variable between fish, there were no significant differences between white or opaque cells at any of the temperatures tested (Figure 4D). Interestingly, whereas white and opaque cells generally maintained their phenotype after dissemination, we did observe a significantly elevated level (~18%, *p* < 0.01) of white-to-opaque switching in the tails of fish kept at 25°C (Figure 4E), perhaps indicating that this niche promotes switching to the opaque state. In general, however, we conclude that the differences in virulence between white and opaque cells are not due to differences in dissemination by the two cell types.

### Comparative analysis of filamentation and growth rates in white and opaque cells

**Figure 5:**
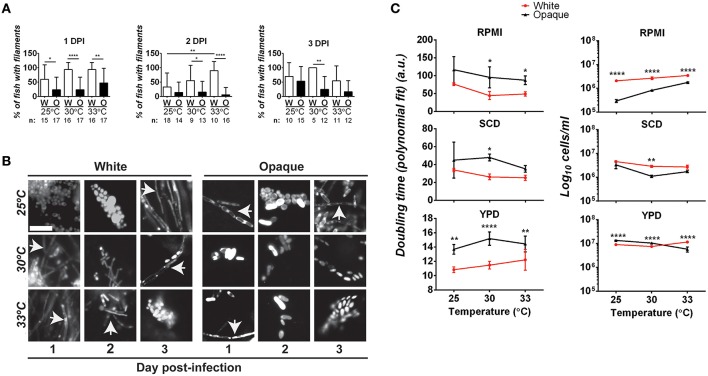
**Comparative analysis of filamentation and growth rates in white and opaque ***C. albicans*** cells. (A)** Zebrafish larvae were infected with 21–50 AlexaFluor-568 stained *C. albicans* white (W) or opaque (O) cells expressing dTomato and housed at the indicated temperature after infection. At 1, 2, and 3 days post-infection (DPI) live fish were crushed (see Methods) and assessed for the presence of filaments microscopically. “n” indicates the number of fish examined for each experiment. Shown are the mean percentages of fish containing filamentous *C. albicans* cells ± SD. ^*^p < 0.05; ^**^p < 0.01; ^****^p < 0.0001. Data are a compilation of five independent experiments. **(B)** Representative microscopic images of the fish assessed in **(A)**. Arrows indicate filaments/hyphae. Magnification, 40 ×; scale bar, 20 μm. **(C)** *C. albicans* white (CAY4975) and opaque (CAY4986) cells were grown in 96-well plates in YPD, SCD, or RPMI media at 25, 30, or 33°C. Cell growth was analyzed using a Biotek Synergy HT plate reader for 24 h. OD_600_ was measured at 15 min intervals. Data were analyzed using a previously described Matlab script. Shown are the average Log_10_ cells/ml at saturation ± SD for white and opaque cells at the designated temperature. Data is representative of 3–4 biological replicates each done in triplicate. Statistically significant differences were determined by two-way ANOVA and Sidak's Multiple Comparisons test. ^*^p < 0.05; ^**^p < 0.01; ^***^p < 0.001; ^****^p < 0.0001.

The ability to transition between yeast and hyphal forms is closely linked to virulence in *C. albicans* white cells (Sudbery, 2011). We therefore examined whether differences in filamentation may account for virulence differences between white and opaque cells. To do this, fish were infected with 21–50 *C. albicans* cells and at 1, 2, and 3 days post-infection fish were examined microscopically for the presence of filamentous cells (see **Methods**). At one day post-infection, 60–94% of fish infected with white cells (at 25, 30, or 33°C) showed evidence of filamentation (Figures 5A,B). Filaments were also present in fish infected with opaque cells at all three temperatures. However, only 23–47% of fish infected with opaque cells showed evidence of filamentation, significantly less than that of fish infected with white cells (Figures 5A,B). Filamentation increased with temperature for fish infected with both white and opaque cells. At 25°C, 60% of white cell-infected fish and 23% of opaque-cell infected fish contained filamentous cells, whereas at 33°C, 94% of white cell-infected fish and 47% of opaque cell-infected fish showed filamentation (Figures 5A,B). At 2 and 3 days post-infection, fish infected with white cells continued to show significantly more filamentation than those infected with opaque cells (Figures 5A,B). We note that in all fish examined yeast cells were present in addition to any filamentous cells. Representative images for infection with each cell type and at different time points are shown in Figure 5B. These data establish that white cells undergo filamentation more readily than opaque cells during zebrafish infection, and this could be one factor contributing to the increased virulence of white cells at higher temperatures.

To further examine the properties of white/opaque cells that may impact virulence, we compared the growth rates of the two cell types under *in vitro* culture conditions. Doubling time and cell concentration at saturation were assessed at different temperatures and for cells grown in YPD, SCD, or RPMI media. At the completion of each assay, cells were also examined for phenotypic state and filamentation. We found that 100% of white cells and >95% of opaque cells retained their phenotypic state throughout the course of these assays. In general, white cells grew as fast, if not faster, than opaque cells under each of the *in vitro* conditions tested, and also reached a higher cell density at saturation (Figure 5C). In both RPMI and SCD media, white cells also grew faster at the elevated temperatures (30 and 33°C) than at 25°C, whereas opaque cells did not show a clear trend with changes in temperature. Filamentation was not observed when white or opaque cells were grown in SCD or YPD media at any of the temperatures tested. In contrast, white cells grown in RPMI medium exhibited filamentation and opaque cells formed chains of cells in this medium, and both of these phenotypes were strongest at elevated temperatures (see Figure S2).

This data indicates that white cells are generally fitter than opaque cells under several standard *in vitro* culture conditions. The faster growth rates of white cells at higher temperatures may contribute to the increased virulence of these cell types in our zebrafish assays. However, growth rates alone cannot explain the higher virulence of white cells over opaque cells, given that similar CFU numbers are obtained with the two cell types in infection (Figure 4B).

### Filamentous growth in both *C. albicans* white and opaque cells promotes virulence

The yeast-to-hyphal switch is an established virulence factor in *C. albicans*, at least for cells in the white state (Sudbery, 2011). The role of filamentation was previously examined in the zebrafish model, and white cells that were unable to form hyphal filaments found to be defective in virulence (Brothers et al., 2011). We therefore evaluated whether filamentation contributes to the virulence of opaque cells in zebrafish. *C. albicans* strains were constructed that were lacking *EFG1* and *CPH1* genes, resulting in strains that are unable to form hyphal cells (Brothers et al., 2011; Si et al., 2013). We assessed whether opaque cells lacking *EFG1/CPH1* formed filaments *in vivo*; fish were housed at 25, 30, or 33°C, and after 1, 2, or 3 days of infection fish were sacrificed and assessed microscopically for the presence of filaments (see Materials and Methods). No filaments were observed in any of the infected fish (see Figure S3), demonstrating that this strain does not undergo filamentation *in vivo*.

**Figure 6:**
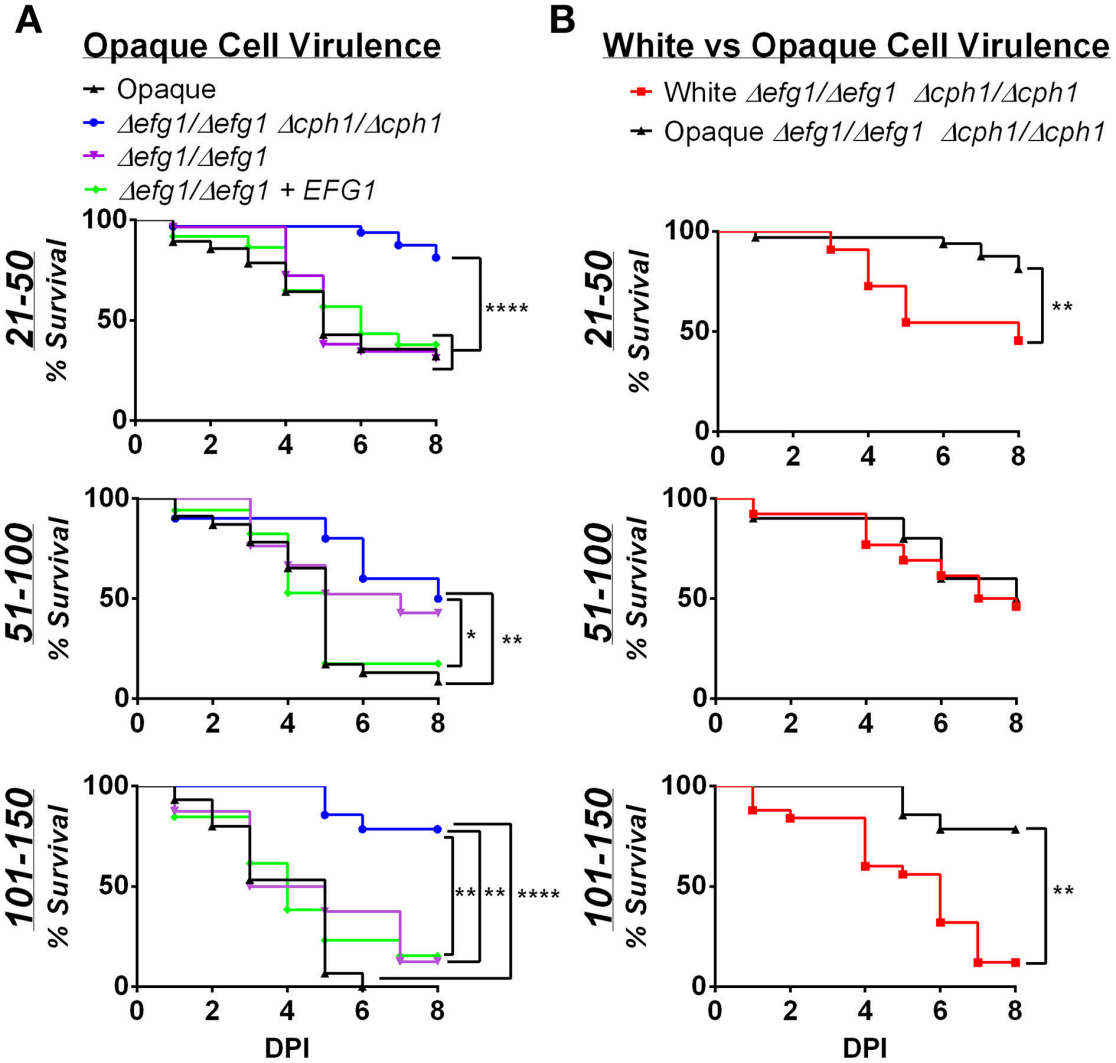
**Virulence is reduced in filamentation-deficient white and opaque cells**. Zebrafish larvae were infected with the indicated number of *C. albicans* cells and kept at 30°C. **(A)** shows survival data comparing infection with different opaque strains. CAY4986 (control opaque), CAY1579 (opaque Δ*efg1/*Δ*efg1* Δ*cph1/*Δ*cph1*), CAY3292 (opaque Δ*efg1/*Δ*efg1*), and CAY4384 (opaque Δ*efg1/*Δ*efg1* + *EFG1*). **(B)** compares the virulence of a white Δ*efg1/*Δ*efg1* Δ*cph1/*Δ*cph1* strain (CAY6597) with that of the equivalent opaque strain (CAY1579). Note that the survival curves for the CAY1579 strain are the same for both **(A,B)**. 8–37 fish were infected for each group in each inoculum range. Data are pooled from 7 individual experiments. Statistically significant differences were determined by Log rank test. ^*^p < 0.05; ^**^p < 0.01; ^****^p < 0.0001.

We next infected zebrafish with *C. albicans* white or opaque cells over a range of inocula and monitored fish daily for survival at 30°C. Infection with opaque cells lacking *EFG1*/*CPH1* resulted in significantly less killing than wildtype opaque cells at each of the inocula tested (Figure 6A). For example, using an inoculum of 21–50 cells, more than 50% of the larvae were killed by wildtype opaque cells by day 5, whereas only 23% of the larvae succumbed to infection with the mutant strain after 8 days of infection. We also compared the virulence of these strains with an *efg1/efg1* mutant and an *EFG1*-complemented version of this strain. The *efg1/efg1* mutant was significantly more virulent than the *efg1/efg1 cph1/cph1* double mutant when using an inoculum of 21–50 or 101–150 cells (Figure 6A). Together, these data indicate that *CPH1* and *EFG1* increase the virulence of *C. albicans* opaque cells.

We also compared the virulence of *efg1/efg1 cph1/cph1* mutants between white and opaque cells. In two inoculum ranges (21–50 and 101–150 cells) the opaque mutant was significantly less virulent than the white mutant (Figure 6B). This provides further evidence that differences in virulence between white and opaque cells cannot simply be due to differences in the ability of these cells to undergo filamentation *in vivo*.

### *C. albicans* white cells are preferentially phagocytosed by innate immune cells compared to opaque cells

Differences in virulence between white and opaque cells may be due, at least in part, to differential interactions with host immune cells. Previous studies showed that the two cell states interact differentially with several immune cell types and that these interactions are dependent on the environment (e.g., 2D vs. 3D) in which the experiments were performed (Kolotila and Diamond, 1990; Geiger et al., 2004; Lohse and Johnson, 2008; Sasse et al., 2013).

**Figure 7:**
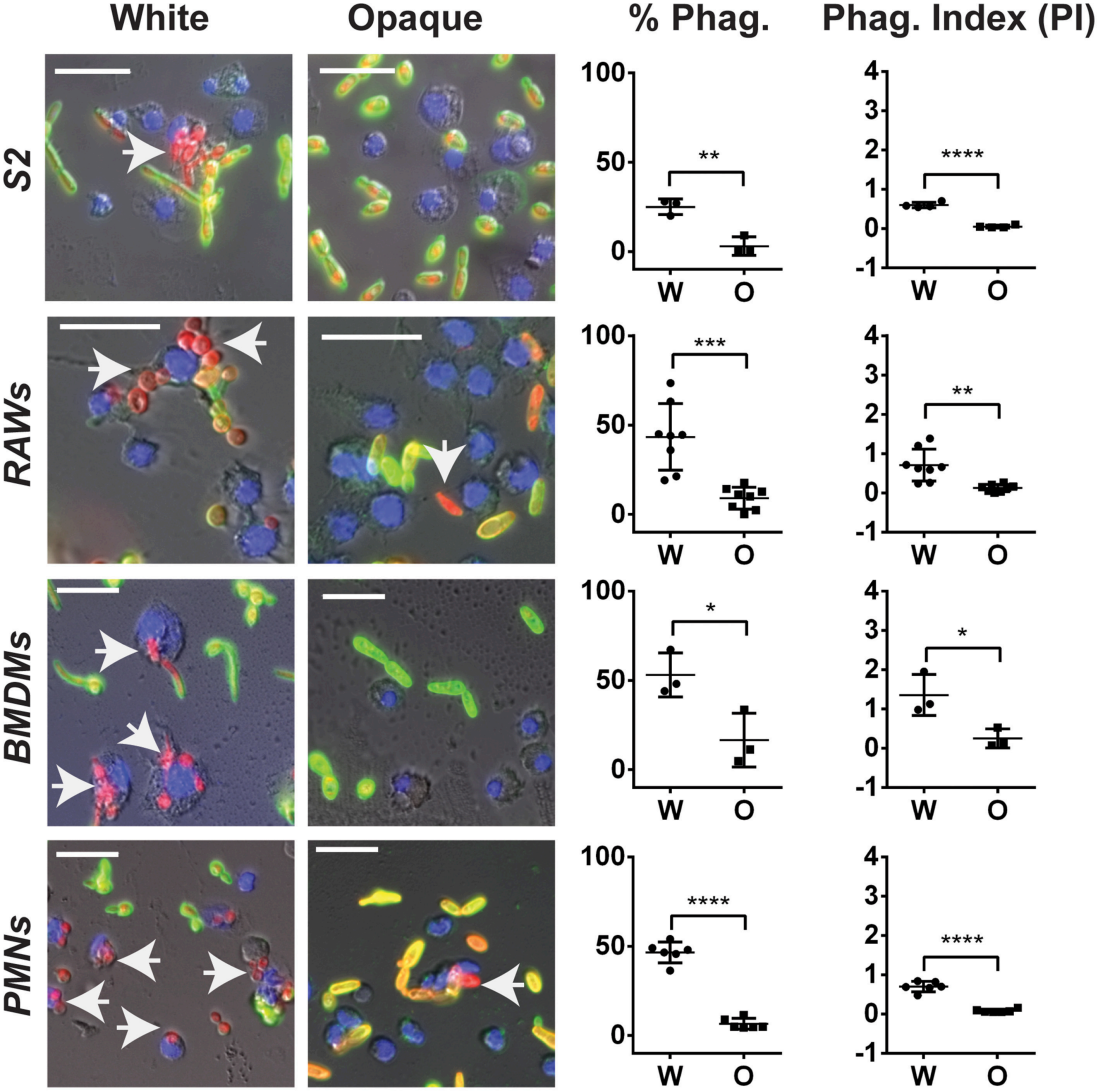
*****C. albicans*** white cells are phagocytosed more efficiently than opaque cells by macrophages ***in vitro*****. The indicated immune cell type was incubated with *C. albicans* white (CAY4975) or opaque (CAY4986) cells for 30 min to 2 h (see Methods) at 28°C (S2 cells) or 37°C (RAWs, BMDMs, and PMNs) and imaged by fluorescence microscopy. Blue (Hoechst) staining represents macrophage nuclei, internalized *C. albicans* appear red (dTomato label), and extracellular *C. albicans* are stained both green (using an anti-*C. albicans* antibody) and red. Arrows point to phagocytosed cells. The percent phagocytosis (total number of macrophages that have phagocytosed ≥ 1 *C. albicans* cell divided by the total number of macrophages scored) and phagocytic index (PI) (total number of *C. albicans* cells phagocytosed divided by the total macrophages scored) were quantified for both white (W) and opaque (O) cells. Data is represented as the mean ± SD and each dot represents a single biological replicate that was assayed in triplicate. At least 100 macrophages were quantified per assay. A Student's *t*-test was performed to determine statistically significant differences between the two groups. ^*^p < 0.05; ^**^p < 0.01; ^***^p < 0.001; and ^****^p < 0.0001. Arrows represent internalized *C. albicans*. Scale bars, 20 μm.

We first re-examined the differences between white and opaque cells in their *in vitro* interactions with both macrophages and neutrophils. White and opaque cells were co-incubated with three different macrophage cell types (*Drosophila* S2 cells, murine RAW264.7 cells, and primary mouse bone marrow derived macrophages (BMDMs)), as well as human neutrophils (PMNs), and phagocytosis frequencies were determined by microscopy. For this analysis, *C. albicans* cells expressing a dTomato reporter were used and non-phagocytosed cells were detected by staining with an anti-*Candida* antibody that did not penetrate immune cells (see Methods). Using this approach, internalized *C. albicans* cells appear red, while those that are not phagocytosed are both green and red (Figure 7). Phagocytosis was determined after a 30 min co-incubation with PMNs, a 1 h co-incubation with RAW and BMDM cells, or a 2 h co-incubation with S2 cells. We note that white and opaque cells generally remained in the yeast state, although a minority of white cells began to filament by the end of the assays, especially when performed at 37°C.

Using these assays, each immune cell type preferentially phagocytosed *C. albicans* white cells over opaque cells. The percent of immune cells that had phagocytosed white cells was 25, 43, 53, and 47% using S2, RAW, BMDM, and PMN cells, respectively, whereas the percent of the same immune cells that had phagocytosed opaque cells was only 3, 9, 17, and 7%, respectively (Figure 7). This demonstrated that white cells are preferentially phagocytosed over opaque cells by 8-, 5-, 3-, and 7-fold for S2, RAW, BMDM, and PMN cells, respectively. In addition to differences in the percentages of immune cells that had phagocytosed white vs. opaque cells, the total number of *C. albicans* cells that had been phagocytosed by each immune cell type was examined to define the phagocytic index (PI). This value incorporates both differences in the number of immune cells that had taken up at least one *C. albicans* cell and the average number of *C. albicans* cells phagocytosed per immune cell, and may more accurately reflect differences in the efficiency of phagocytosis (Lohse and Johnson, 2008). The PI was also significantly higher for white cells than opaque cells with each immune cell type (Figure 7).

Together, these results establish that both macrophages and neutrophils preferentially phagocytose *C. albicans* white cells over opaque cells *in vitro*. For S2 and RAW cells our data corroborate that of Lohse and Johnson (Lohse and Johnson, 2008), while Sasse et al. also observed that human neutrophils preferentially phagocytose white cells over opaque cells, at least when analyzed on a glass slide or in the wells of glass-bottom plates (Sasse et al., 2013). We established that the preferential phagocytosis was not specific to strain background, as macrophages and neutrophils also preferentially phagocytosed white cells over opaque cells from other *C. albicans* strain backgrounds (Figures S4A,B). This data establishes that multiple immune subtypes, from *Drosophila* to mammals, preferentially phagocytose *C. albicans* white cells over opaque cells when co-cultured *in vitro*.

### Immune cells differentially phagocytose *C. albicans* white and opaque cells *in vivo*

**Figure 8:**
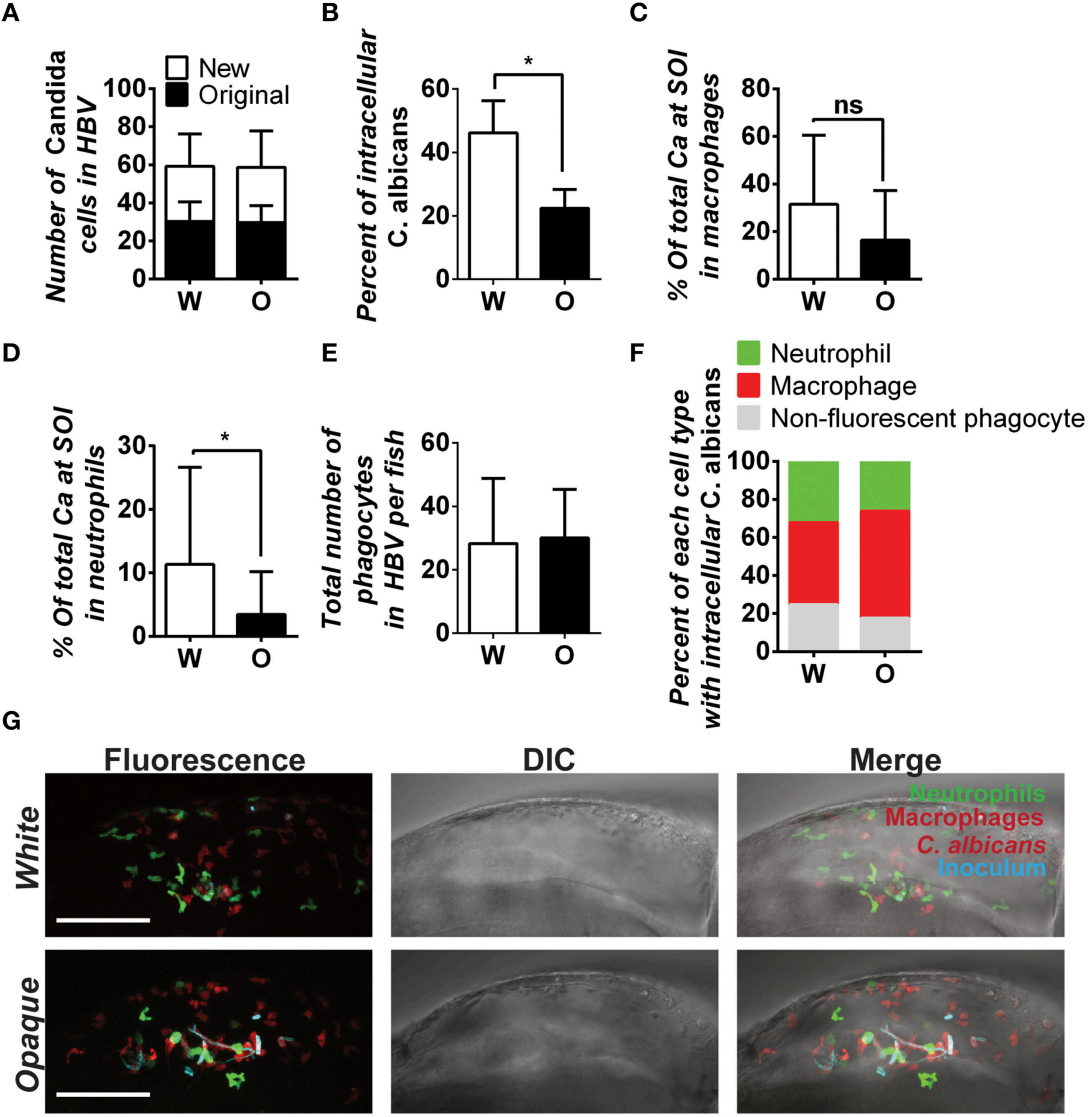
*****C. albicans*** white cells are phagocytosed more efficiently than opaque cells *in vivo***. AlexaFluor-647 stained white or opaque cells expressing dTomato (CAY4975 and CAY4986, respectively) were microinjected into the hindbrain ventricle of transgenic fish that contain EGFP-expressing neutrophils and mCherry-expressing macrophages. Larvae were screened post-injection to identify those with 20–50 *C. albicans* cells at the site of injection and subsequently imaged 4 h post-injection via confocal microscopy. **(A)** The number of *C. albicans* cells present in the hindbrain ventricle (HBV) after microinjection (AlexaFluor 647-labeled cells) and the number of *C. albicans* cells present 4 h post-injection (cells displaying cytosolic dTomato fluorescence) ± SD. **(B)** The percentage of *C. albicans* cells inside vs. outside phagocytes at the site of infection was calculated for each fish (see Methods). Shown are the average percentages of intracellular cells ± SEM. Statistically significant differences were determined using a Student's *T*-test. ^*^p < 0.05. **(C)** Mean percentages of *C. albicans* cells present in zebrafish macrophages at the site of infection (SOI) ± SD. Statistically significant differences determined using a Student's *T*-test. ns, not significant. **(D)** Mean percentages of *C. albicans* cells present in neutrophils at the site of infection ± SD. Statistically significant differences determined using a Student's *T*-test. ^*^p < 0.05. **(E)** Total number of phagocytes (neutrophils, macrophages, and non-fluorescent phagocytes) recruited to the site of infection for each fish. Shown are mean numbers of phagocytes in the HBV per fish ± SD. **(F)** Breakdown of phagocyte subtypes with intracellular *C. albicans* cells at the site of infection. **(G)** Confocal images of representative fish in white and opaque cell cohorts. Maximum projections made from Z-stacks with 39 slices and 23 slices for white and opaque cells, respectively. Scale bar, 100 μm. Data were collected over 9 independent experiments with 14 and 22 individual fish in the white and opaque cohorts, respectively.

Phagocytosis by immune cells is dependent on the environment, and *in vitro* assays may not reflect *in vivo* interactions with phagocytes (Sasse et al., 2013). To assess the interaction of white and opaque cells with immune cells *in vivo*, we utilized transgenic zebrafish containing EGFP-expressing neutrophils and mCherry-expressing macrophages. We note that due to mosaic silencing of the UAS promoter, a subset of zebrafish macrophages do not express mCherry. *C. albicans* cells were injected into the hindbrain (inoculum of 20–50 cells) and imaged at 4 h post-infection (Figure 8A). These experiments assessed both the recruitment of immune cells to the hindbrain injection site and the number of internalized *C. albicans* cells. Overall, internalization of white cells was more efficient than opaque cells, as approximately 46% of white cells were found inside zebrafish phagocytes, whereas only 22% of opaque cells were internalized (Figure 8B). Notably, the total number of fungal cells in the hindbrain ventricle (HBV) of infected fish was similar for both white and opaque cells (Figure 8A), indicating that the greater containment of white cells was not simply due to differences in the number of fungal cells.

We next determined immune cell type-specific phagocytosis of white and opaque cells using the dual-reporter neutrophil and macrophage zebrafish line. Macrophages preferentially phagocytized white cells over opaque cells *in vivo*, with phagocytosis of 31.5% of white cells and 16.4% of opaque cells, although this difference was not significant (Figure 8C). Neutrophils also preferentially phagocytized white cells over opaque cells *in vivo*. Neutrophils phagocytized 11.3% of white cells, whereas only 3.4% of opaque cells were taken up by this cell type (*p* < 0.05, Figure 8D). The differential phagocytosis of white and opaque cells was not due to increased recruitment of macrophages and neutrophils to the site of infection, as the number of fluorescent phagocytes present in the hindbrain ventricle was similar in fish infected with either cell type (Figures 8E–G; Movies S3, S4). Taken together, these data demonstrate that *C. albicans* opaque cells escape immune containment in the vertebrate host more frequently than white cells. We discuss the potential consequences of differential phagocytosis for virulence in the host below.

## Discussion

In this study, we exploit zebrafish larvae as a model to study *C. albicans* white and opaque cell states during an *in vivo* infection. Zebrafish larvae can be maintained at a wide range of temperatures, enabling us to determine the effect of temperature on virulence in a vertebrate host. They are also transparent, so that immune cell recruitment, as well as *C. albicans* dissemination and filamentation, can be examined non-invasively. We show that both white and opaque cell types infect and disseminate within the zebrafish, ultimately resulting in death of the host. These results establish that opaque cells, as well as white cells, are capable of a systemic infection that kills the host. Interestingly, the virulence of white and opaque cells was similar at 25°C, but white cells were significantly more virulent than opaque cells at higher temperatures (30 or 33°C), consistent with studies that compared the two cell types in a mouse tail vein model of systemic candidiasis (Kvaal et al., 1997). Our results establish that temperature plays a hitherto unrecognized role in determining the relative virulence of white and opaque cells. Differences in white and opaque fungal burdens did not account for differences in virulence, as CFUs were similar for both cell types at each temperature and throughout the course of the infection. This is despite the fact that white cells generally grew faster and reached a higher cell density than opaque cells when cultured under a variety of *in vitro* conditions.

In addition to temperature-dependent differences in virulence between white and opaque cells, we found that the virulence of *both* cell types increased as the temperature of the infection was raised from 25 to 33°C. Thus, elevated temperatures enhance the virulence of *C. albicans* cells independent of the phenotypic state. Many pathogenic fungi are thermally dimorphic, switching between yeast and filamentous forms when grown at lower (e.g., 25°C) or higher (37°C) temperatures, and these transitions play a central role in promoting pathogenesis (Kumamoto and Vinces, 2005; Sudbery, 2011; Gow et al., 2012). In *C. albicans*, white cells usually grow in the yeast state at ambient temperatures but are induced to undergo filamentation at 37°C (Sudbery, 2011; Shapiro et al., 2012). In contrast, *C. albicans* opaque cells exhibit optimal filamentation at 25°C *in vitro*, and raising the temperature reduces filamentous growth (Si et al., 2013). In the zebrafish model, we found that both white and opaque cells underwent filamentation over the range of temperatures tested, indicating that *in vivo* signals induce filamentation in both cell types and at multiple temperatures. Notably, the percentage of fish with observable filaments was significantly higher in fish infected with white cells than those infected with opaque cells. It is therefore possible that increased filamentation contributes to the increased virulence of white cells relative to opaque cells at higher temperatures. However, fish infected with white cells also showed increased filamentation relative to opaque cells at 25°C where there was no difference in virulence, suggesting that enhanced filamentation alone cannot account for the elevated virulence of white cells compared to opaque cells at 30 or 33°C.

Temperature could impact virulence by several mechanisms other than induction of the yeast-hyphal switch. *C. albicans* growth rates are influenced by temperature, and both white and opaque cells generally exhibit faster growth rates at 30 or 33°C than at 25°C, at least under standard *in vitro* conditions, which could contribute to increased virulence at higher temperatures. We note, however, that CFUs were similar when compared at 2 days between infections performed at different temperatures (Figure 4B). Thermal signals also regulate the expression of virulence genes in several species, such as bacteria that alternate between a vector or environmental reservoir and a mammalian host (Konkel and Tilly, 2000; Réjasse et al., 2012). By analogy, virulence genes could similarly be expressed at higher levels in *C. albicans* cells grown at elevated temperatures. Alternatively, differences in *C. albicans* virulence could be due to temperature-dependent differences in the host immune system. For example, it was previously shown that lowering the temperature reduces the immune response in fish (Le Morvan et al., 1998). It is therefore possible that stronger immune responses at the higher temperatures increase *C. albicans* virulence in zebrafish larvae, as hyper-inflammation could increase pathogenesis due to immunopathology (Lionakis et al., 2012; Majer et al., 2012). Future experiments could address this hypothesis by using chemical inhibitors of neutrophil recruitment (Deng et al., 2013) and evaluating how this alters *C. albicans* virulence at different temperatures.

To further examine the role of filamentation in infection, we utilized *C. albicans efg1*Δ*/*Δ *cph1*Δ*/*Δ mutants that were unable to form hyphae under a wide range of *in vitro* conditions (Stoldt et al., 1997; Brown et al., 1999; Sharkey et al., 1999; Braun and Johnson, 2000; Si et al., 2013). Brothers et al. previously showed that *efg1*Δ*/*Δ *cph1*Δ*/*Δ cells in the white state were unable to form true hyphae during infection of zebrafish larvae (Brothers et al., 2011). These mutants also exhibited reduced killing, as well as reduced fungal burdens, compared to wildtype cells. In this study we compared wildtype and *efg1*Δ*/*Δ *cph1*Δ*/*Δ cells in both white and opaque states, and found that mutant cells were significantly reduced for virulence in both phenotypic states. This result indicates that the filamentation program (or genes co-regulated with the filamentation program) promotes virulence in both white and opaque cells. Interestingly, we found that white *efg1*Δ*/*Δ *cph1*Δ*/*Δ mutants still exhibited greater virulence than isogenic opaque mutants, again implying that factors other than the formation of hyphae distinguish these cell types *in vivo*.

Our studies also addressed the stability of white and opaque states during infection. Opaque cells have been shown to be highly temperature sensitive, often switching to the white state *en masse* when grown at 37°C *in vitro* or during systemic candidiasis in the mouse tail vein model (Slutsky et al., 1987; Rikkerink et al., 1988; Srikantha and Soll, 1993; Kvaal et al., 1997). However, environmental cues can stabilize opaque cells at 37°C, including N-acetylglucosamine, CO_2_ and anaerobiasis (Dumitru et al., 2007; Ramírez-Zavala et al., 2008; Huang et al., 2009, 2010), and white-to-opaque switching was observed in one clinical isolate during passage through the mammalian intestinal tract (Ramírez-Zavala et al., 2008). Our experiments demonstrate that both white and opaque cell types are stably maintained during infection of zebrafish larvae, independent of the temperature at which the experiment is performed. Thus, whereas opaque cells were unstable when cultured at 33°C *in vitro*, switching back to the white state *en masse*, these cells were stably maintained during infections performed at 33°C, even out to 7 days post-infection (Figure 1 and Figure S1). This surprising result reveals that the environment in the zebrafish host is generally supportive of maintaining the opaque state. It is currently unknown as to the exact nature of the *in vivo* signal(s) that regulate opaque cell stability, although the zebrafish model makes this an attractive system for further investigation of this phenomenon.

Finally, we performed a detailed analysis on the interaction between *C. albicans* cells and immune cells. This compared differences both *in vitro* and *in vivo*, as conditions in the host have been shown to influence the phagocytosis of fungal cells, as well as the fate of these cells once inside the immune cell (Calderone and Sturtevant, 1994; Rubin-Bejerano et al., 2003; Lorenz et al., 2004; Kumamoto and Vinces, 2005; Frohner et al., 2009; Brothers et al., 2011, 2013). Our studies support previous *in vitro* findings that white cells are preferentially phagocytosed by both macrophages and neutrophils relative to opaque cells (Lohse and Johnson, 2008; Sasse et al., 2013). Furthermore, we show that white and opaque cells are also differentially phagocytosed during infection, as white cells showed a 3-fold increase in uptake by host neutrophils compared to opaque cells. Previous work in zebrafish indicates that efficient early phagocytosis blocks the yeast-to-hyphal switch in white cells, and can play an important role in limiting invasion and mortality *in vivo* (Brothers et al., 2013). Thus, the efficient uptake of white cells may limit their virulence, which would be even more pronounced without high rates of phagocytosis. Alternatively, however, it is possible that greater phagocytosis could contribute to increased virulence by driving a hyper-inflammatory response in the host. Studies in mice have established that some arms of the innate immune response result in immunopathology during systemic Candidiasis. Thus, loss of the chemokine receptor, Ccr1, or the type I interferon receptor subunit, IFNAR1, have been shown to protect against fatal fungal disease in the mouse (Lionakis et al., 2012; Majer et al., 2012). Future experiments will address whether the preferential phagocytosis of white cells relative to opaque cells results in increased or decreased pathogenesis in zebrafish.

Differential phagocytosis of white and opaque cells is likely due to differences in pathogen-associated molecular patterns (PAMPs) associated with the two cell types. We note that differences were observed using phagocytes from diverse hosts (*Drosophila*, zebrafish, mouse, and human), even though these organisms often utilize distinct pattern-recognition receptors (PRRs). To date, the specific PRRs on zebrafish macrophages and neutrophils that recognize fungal cells have not been identified, although both TLRs and NOD-like receptors are present (van der Vaart et al., 2012). Fungal PAMPs that mediate uptake by zebrafish phagocytes are likely to include cell wall ligands such as mannan, glucan and chitin, which have been shown to mediate host-pathogen interactions in vertebrate species (Yang et al., 2014; Erwig and Gow, 2016). A detailed characterization of the cell wall and secretomes of white and opaque cells could therefore provide insights into the differential recognition of these two cell types by innate immune cells, as well as the contribution of these factors to virulence of the two cell states in *C. albicans*.

## Author contributions

EM, RB, RW, and AB made substantial contributions to the conception and design of the work. EM, ZN, and AB performed experiments and EM, AB, RW, and RB analyzed results and interpreted the data. EM, RB, and RW wrote the manuscript and SJ and RC edited the manuscript. RC and SJ provided the zebrafish facility and also offered technical assistance. KB designed the zebrafish infection model and offered technical assistance and helped troubleshoot experimental procedures.

### Conflict of interest statement

The authors declare that the research was conducted in the absence of any commercial or financial relationships that could be construed as a potential conflict of interest.
