# Supplementary figures and images for: Phenotypic Plasticity Regulates Candida albicans Interactions and Virulence in the Vertebrate Host
Source: Front Microbiol. 2016 May 26;7:780. doi: 10.3389/fmicb.2016.00780 (PMC4880793; doi:10.3389/fmicb.2016.00780)

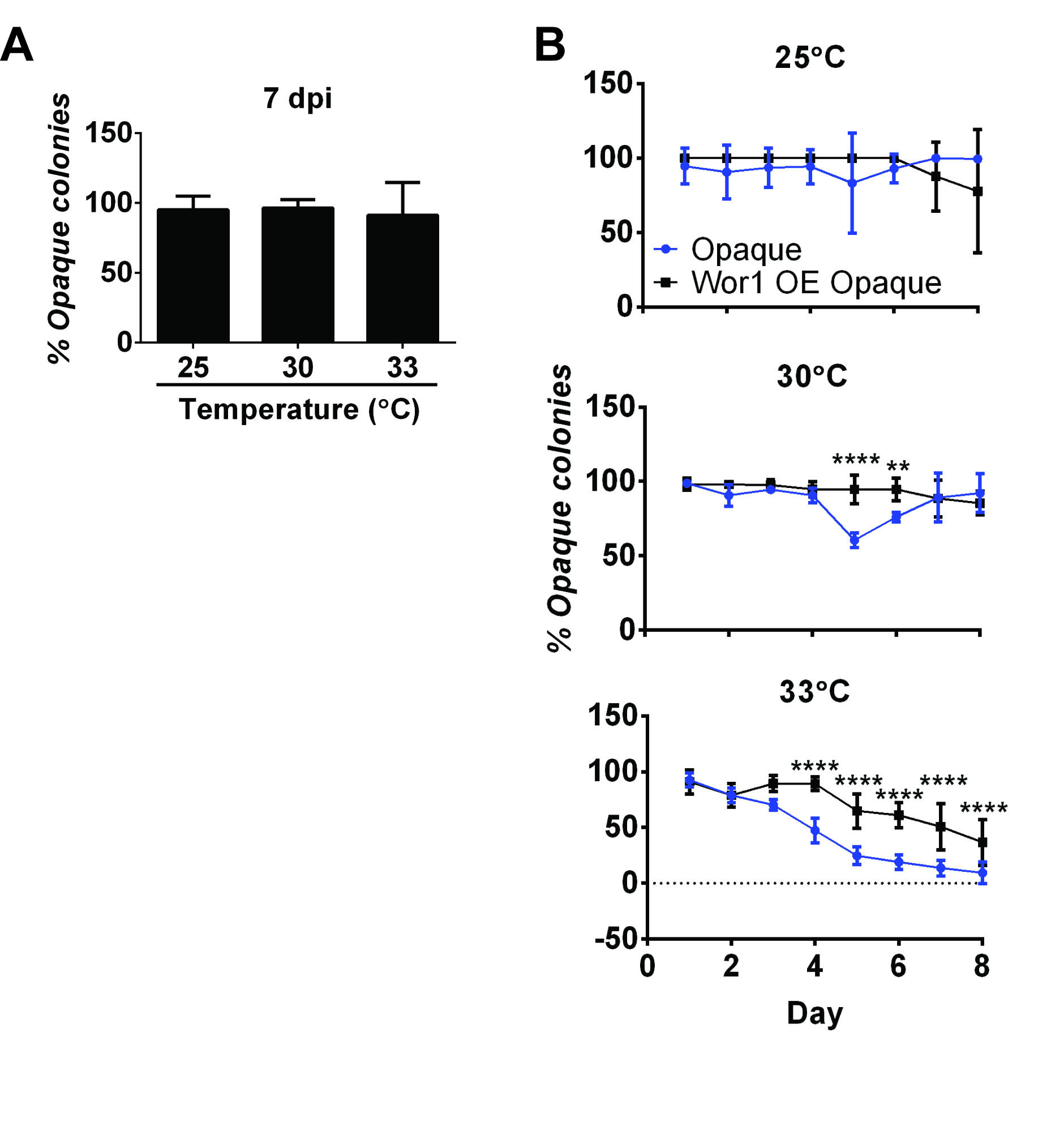

Supplement: Supplementary file 1 [file Image1.TIF]

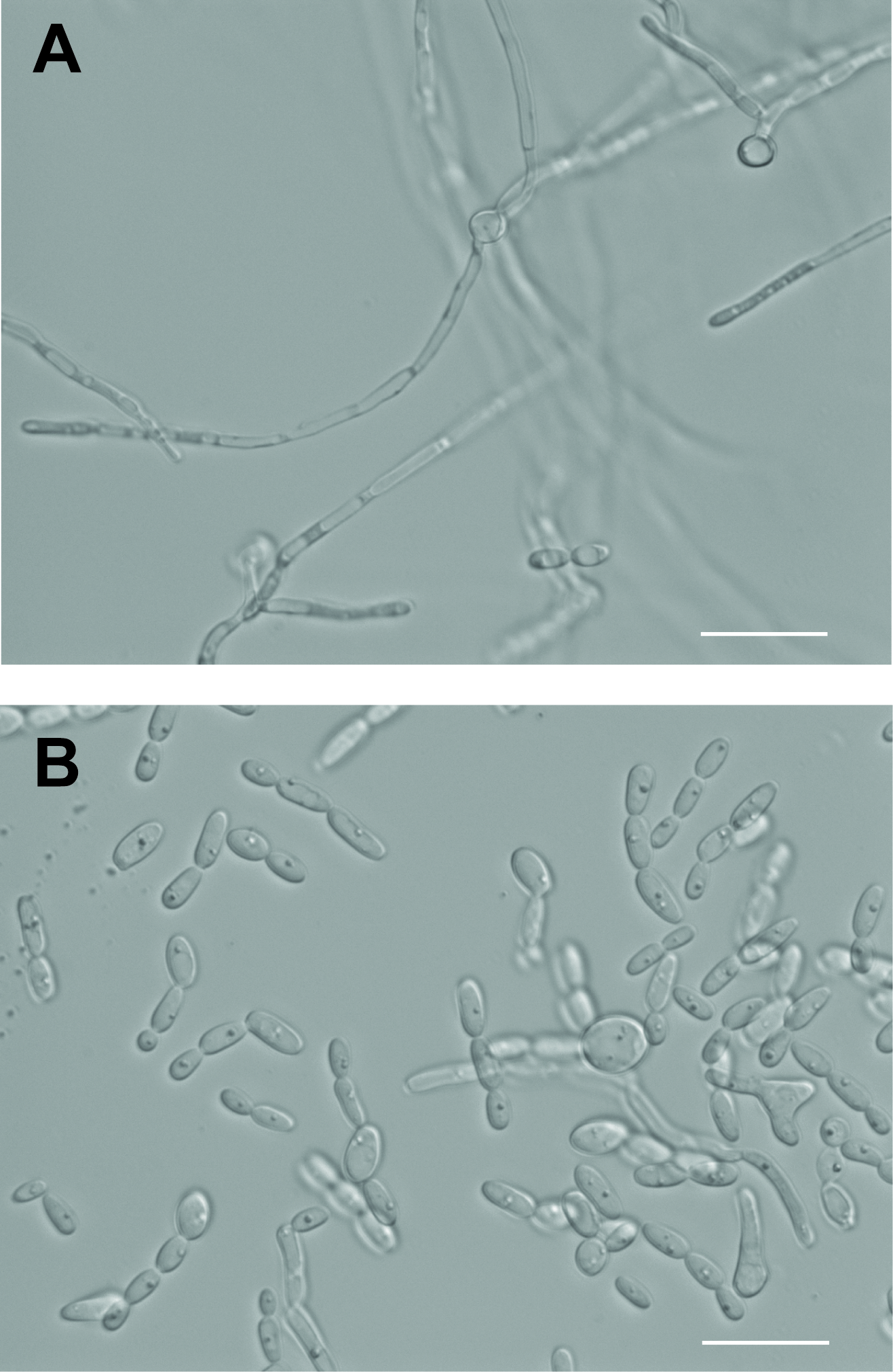

Supplement: Supplementary file 2 [file Image2.TIF]

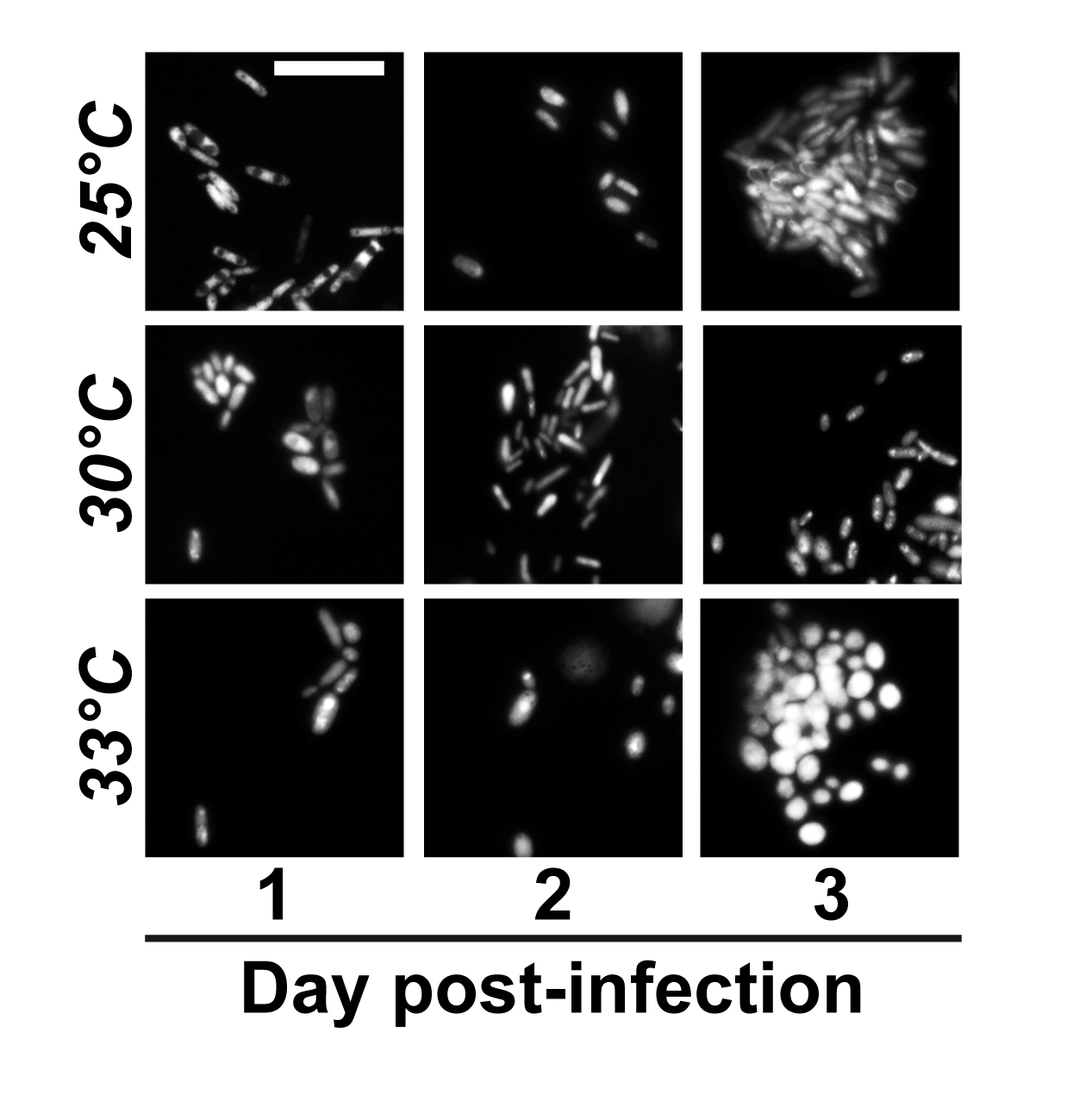

Supplement: Supplementary file 3 [file Image3.TIF]

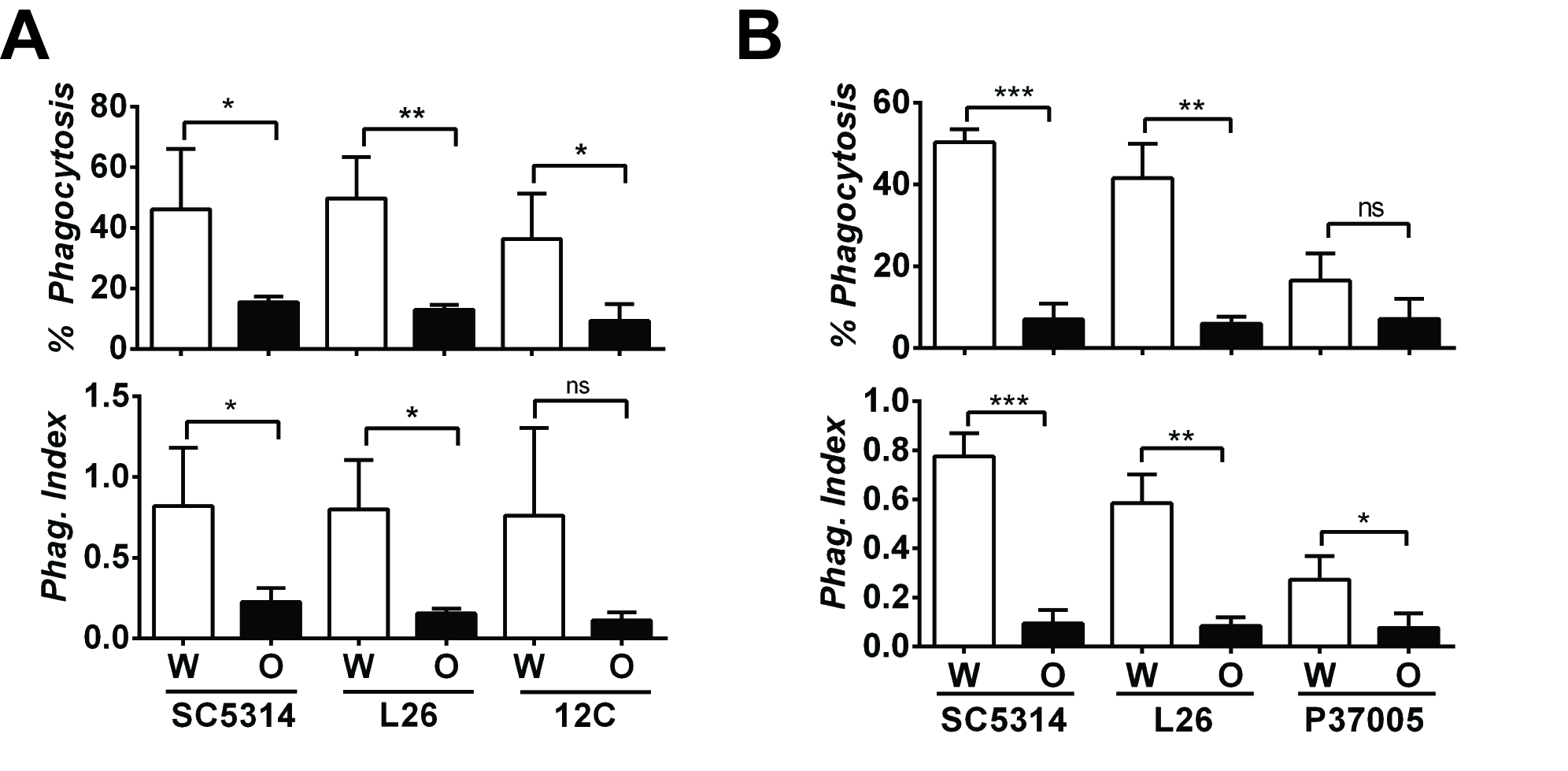

Supplement: Supplementary file 4 [file Image4.TIF]
