# Supplementary material for: Phenotypic Plasticity Regulates Candida albicans Interactions and Virulence in the Vertebrate Host
Source: Front Microbiol. 2016 May 26;7:780. doi: 10.3389/fmicb.2016.00780 (PMC4880793; doi:10.3389/fmicb.2016.00780)
Supplement: Supplementary file 5 [file Table1.DOCX]

**Table S1.**

| Day post-infection | Temperature (°C) | % Opaque colonies ± SD | n* |
| --- | --- | --- | --- |
| 1 | 25 | 100 ± 0.0 | 2 |
| 2 | 25 | 100 ± 0.0 | 2 |
| 3 | 25 | 100 ± 0.0 | 9 |
| 5 | 25 | 98.1 ± 6.6 | 12 |
| 7 | 25 | 100 ± 0.0 | 12 |
| 11 | 25 | 79.2 ± 35.1 | 12 |
| 12 | 25 | 97.4 ± 4.0 | 14 |
| 1 | 30 | 99.0 ± 3.2 | 98 |
| 2 | 30 | 95.3 ± 16.5 | 21 |
| 3 | 30 | 100 ± 0.0 | 2 |
| 4 | 30 | 94.8 ± 16.1 | 34 |
| 5 | 30 | 95.9 ± 20.0 | 25 |
| 7 | 30 | 100 ± 0.0 | 13 |
| 8 | 30 | 73.3 ± 48.9 | 4 |
| 3 | 33 | 98.2 ± 4.2 | 17 |
| 5 | 33 | 99.2 ± 2.3 | 9 |
| 7 | 33 | 100 ± 0.0 | 13 |

Fish were infected with *C. albicans* opaque cells (CAY4986, expressing *pACT1-WOR1*) and kept at 25, 30, or 33ºC after infection. At the indicated time, individual zebrafish were homogenized and plated for CFUs. The total numbers of white and opaque colonies were calculated and shown are the average percent opaque colonies ± SD. Data are a compilation of 20 experiments.

*n, number of fish per group.
