# Supplementary material for: Phenotypic Plasticity Regulates Candida albicans Interactions and Virulence in the Vertebrate Host
Source: Front Microbiol. 2016 May 26;7:780. doi: 10.3389/fmicb.2016.00780 (PMC4880793; doi:10.3389/fmicb.2016.00780)
Supplement: Supplementary file 6 [file DataSheet1.DOCX]

**Supplemental Figure Legends**

**Figure S1. Stability of opaque cells *in vivo* and *in vitro*. (A)** Wild type zebrafish in the Prim25 stage were infected with 2-100 CFU of opaque cells (CAY5202) and kept at 25, 30, or 33°C after infection. At 7 days post infection, individual fish were homogenized and serial dilutions plated onto SCD. After 7 days, the percentages of white and opaque colonies were counted. 15-19 live fish were homogenized for each temperature. Shown are the percentages of opaque colonies at each temperature ± SD. **(B)** Control opaque cells (RBY731) and opaque cells containing *pACT1-WOR1* (CAY4986; Wor1 OE strain) were grown in SCD medium and kept at 25, 30, or 33°C. Each strain was diluted back daily into SCD and passaged for 8 days at the indicated temperature. Each day 100 colonies were plated onto SCD and 7 days later the percentages of white, opaque, and sectored colonies were counted. Shown are the mean percentages of opaque colonies of five biological replicates at each time point for each temperature ± SD.

**Figure S2. White and opaque cells undergo filamentous growth in RPMI medium at 33°C.** White **(A)** and opaque **(B)** cells were grown in RPMI medium at 33°C overnight and pictures of the cultures were taken at 40x. Scale bar, 20 µm.

**Figure S3. An opaque mutant lacking both *EFG1* and *CPH1* does not undergo hyphal growth *in vivo*.** Fish were infected with 21-50 *C. albicans* strain CAY7071, kept at 25, 30, and 33°C post-infection. At the indicated time point fish were crushed (see Methods) and assessed for filamentation microscopically. 3-9 fish were assessed per temperature at each time point. Magnification**,** 40x; scale bars, 20 µm**.**

**Figure S4. Phagocytosis of *C. albicans* white and opaque cells from different strain backgrounds.** **(A)** RAW 264.7 cells were incubated with the indicated white or opaque cells derived from SC5314 (CAY4975 and CAY4986, respectively), L26 (CAY715 and CAY6548, respectively), 12C (CAY713 and CAY6814, respectively), or P37005 (CAY716 and CAY1477, respectively). Cells were co-incubated for 1 h and the percent phagocytosis and phagocytic index quantified. Shown are the mean ± SD of the average % phagocytosis or phagocytic index of 2-3 biological replicates for 3 compiled individual experiments. **(B)** Human neutrophils were incubated with the indicated *C. albicans* strains for 30 min and the percent phagocytosis and phagocytic index were quantified. Shown are the mean ± SD of the average % phagocytosis or phagocytic index of 2-3 biological replicates for 3 compiled individual experiments.

**Movies S1 and S2. Individual stacks thorough the zebrafish hindbrain of infected fish.** Zebrafish were infected with *C. albicans* strain CAY4975 (white cells) (**Movie 1**) or CAY4986 (opaque cells) (**Movie 2**) and kept at 30ºC after infection. At 1-day post infection, zebrafish were imaged via confocal microscopy. Shown is the representative individual Z series images taken for the fish shown in **Figure 2B** and **2D**. Panel **A** represents the 138 slides through the fish imaged in **Figure 2B** and panel **B** represents the 176 slides though the fish imaged in panel **2D**.

**Movies S3 and S4. Individual stacks thorough the zebrafish hindbrain of infected fish.** Zebrafish were infected and imaged via confocal microscopy as described for Figure 10. Shown are the representative individual Z series images taken for the fish shown in **Figure 10G**. Panel **A** represents the 138 slices through the fish imaged in **Figure 10G (upper row)** and panel **B** represents the 176 slices though the fish imaged in **Figure 10G (bottom row)**.
